# Supplementary material for: Experiences of people with opioid use disorder during the COVID-19 pandemic: A qualitative study
Source: PLoS One. 2021 Jul 29;16(7):e0255396. doi: 10.1371/journal.pone.0255396 (PMC8320992; doi:10.1371/journal.pone.0255396)
Supplement: S1 Table — Thematic codebook used for the qualitative analysis of participants’ opinions and experiences during COVID-19 gathered in interviews. (DOCX) [file pone.0255396.s001.docx]

| S1 Table. Thematic Codebook.  Thematic codebook used for the qualitative analysis of participants’ opinions and experiences during COVID-19 gathered in interviews. | | | | |
| --- | --- | --- | --- | --- |
|  | | | | |
| Theme/Sub-Theme | Code | Description | Coverage | Examples from Transcripts |
| 1. Emotions |  |  |  |  |
| 1.1. Indifference | No impact on life or behavior | References to lack of impact on participant’s life or a lack of behavior change by participant to prevent infection during COVID-19 | 5 | *Yeah, I've pretty much carried on continuing to do what I've been doing. (Participant 3; male, 40 years old)* |
|  | Does not care about or believe COVID-19 | References to not caring about COVID-19 and safety measures or not believing the threat of COVID-19 is real, by participants or others (e.g. COVID-19 simply being a scare tactic by the government) | 11 | *As soon as they announced COVID-19 and I saw the big pandemic that it was creating I thought you know what, I'll be fine. I don't need to worry about this. (Participant 1; male, 31 years old)* |
| 1.2. Other Negative Emotions | Scared/fear | References to participants themselves or others feeling scared or fearful emotions during COVID-19 | 19 | *And I saw that there was on the news in the hospital there's like first outbreak of this new thing in Wuhan and yeah it was pretty scary. … It was like beginning in January and then … at the end of February, everything started to get really, really scary. Like really quick. (Participant 12; female, 37 years old)* |
|  | Anxious/Stressed  /Triggered | References to participants feelings anxious emotions during COVID-19 (e.g. stressed, unsettled) | 9 | *It's been stressful. It's like, triggered PTSD and like stuff like that. (Participant 16; female, 25 years old)* |
|  | Unsure/Uncertain | References to participants feeling unsure or uncertain during COVID-19 | 4 | *I do think that we were all very unsure of what to do, because it kept changing. (Participant 8; female, 30 years old)* |
|  | Tired of COVID-19 | References to participants themselves or others being tired of COVID-19 (e.g. wanting everything to return to the way it was before) | 3 | *I think people are sick of it and they're just over it. (Participant 10; male, 40 years old)* |
|  | Isolated/Lonely | References to feeling isolated or lonely during COVID-19 (oneself or others) | 4 | *I feel a little isolated from not being able to use various services for my addiction. (Participant 7; male, 35 years old)* |
|  | Frustrated | References to participants feeling frustrated during COVID-19 | 4 | *I'm frustrated by it. I want to know how some things that are near and dear to my heart are gonna work now. (Participant 2; male, 48 years old)* |
| 1.3. Positive Emotions | Pride | References to participants feeling proud during COVID-19 | 1 | *I am quite proud of people … if someone's overdosing people still stepped up and saved them. (Participant 2; male, 48 years old)* |
| 2. Drug Use and Safety |  |  |  |  |
| 2.1. Changes in the Safety of Drug Use | Less safe due to COVID-19 measures | References to participants using drugs less safely due to COVID-19 restrictions (e.g. building closures, physical distancing) | 8 | *… because we had to be by ourselves. We weren't allowed to be around each other. So basically forcing us to use alone. (Participant 8; female, 30 years old)* |
|  | More hygienic space and use | References to the promotion of hygienic space and germ awareness during COVID-19 | 2 | *It taught me to be cleaner about it … taking care of myself like the germs and stuff, I use alcohol swipes, all my alcohol swabs, I use a lot of them. (Participant 17; male, 45 years old)* |
|  | New strategies to use more safely | References to participants finding new strategies to use drugs more safely during COVID-19 | 3 | *I use the Lifegaurd app*. So, it's like, it's much easier to use alone and not be worried about not being able to get help. Because if I don't make that one-minute timer, it calls 911. (Participant 9; male, 23 years old)* |
|  | No change in safety of use | References to no changes in a participant’s drug use or safety of use during COVID-19 | 4 | *I think everything across the board is the same. (Participant 10; male, 40 years old)* |
| 2.2 Changes in Drug Use | Change in drug availability or price | References to changes in drug availability or price during COVID-19 leading participants to use different types of drugs (i.e. not their typical drug(s) of choice) | 6 | *Apparently, like they're having a hard time getting stuff in to make … how they used to or whatever. So, there's like a lot of different types of dope out. It's weird. (Participant 8; female, 30 years old)* |
|  | Using drugs more as only drugs are accessible | References to participants using more drugs during COVID-19 due to the lack of accessibility of everything else | 2 | *My life didn't … I mean, it changed in the way that like everything was closed, but like the only thing that was easily accessible was drugs. (Participant 12; female, 37 years old)* |
| 2.3. Changes in Purchasing Habits of Drugs | Buying more drugs to limit social encounters | References to participants purchasing more drugs at a time during COVID-19 to limit the time spent outside or interacting with others | 3 | *I would buy bigger amounts of drugs I guess to be able to stay home longer and not have to go out and get them. (Participant 12; female, 37 years old)* |
|  | Buying more drugs due to new influx of money | References to participants purchasing more drugs at a time during COVID-19 due to the emergency money provided by the government | 1 | *[referring to buying more drugs at a time] Of course … I've got thousands of dollars for free. (Participant 15; male, 31 years old)* |
| 2.4. Pharmaceutical Alternatives of Opioids^1^ | Does not find supply of alternatives to be helpful | References to participants not finding the supply of pharmaceutical alternatives provided during COVID-19 helpful | 2 | *It doesn't make a difference because I'm not a pill person, it's not the same drugs. (Participant 13; female, 33 years old)* |
|  | Finds supply of alternatives to be helpful | References to participant finding the supply of pharmaceutical alternatives provided during COVID-19 helpful | 2 | *… it helped me out. It's helping me stay off fentanyl. (Participant 9; male, 23 years old)* |
| 3. Impact on Community |  |  |  |  |
| 3.1. Change in Access to Care Services | Services slowed or closed | References to care services being slower or closed during COVID-19 | 16 | *A lot of places were shut down and some still are shut down, and a lot of them actually shut down for good. (Participant 18; male, 34 years old)* |
|  | Difficulty accessing housing | References to difficultly accessing housing support during COVID-19 (e.g. inability or difficulty accessing shelters) | 5 | *They don't let you in shelters in COVID-19. (Participant 1; male, 31 years old)* |
| 3.2. Change in Interaction with Care Services | Positive change in the ED | References to changes in the ED during COVID-19 that have led to improved care compared to before COVID-19 (e.g. fewer crowds, faster services) | 3 | *I was actually treated really well, and it went super quick and super smooth and so I was pretty stoked on that cause there was literally no one else there. (Participant 18; male, 34 years old)* |
|  | Less user-friendly due to COVID-19 measures | References to community services being less user-friendly due to COVID-19 measures (e.g. physical distancing, mask wearing) | 14 | *You were limited to the amount of people in the emergency. (Participant 6; male, 52 years old)* |
|  | ED became more unwelcoming | References to the ED being more unwelcoming during COVID-19 compared to before (e.g. more judgmental) | 3 | *I just found people not like taking me seriously. (Participant 16; female, 25 years old)* |
|  | Preference for over the phone appointments | References to participants preferring not to have to go in person for appointments for care services and liking over the phone appointments | 2 | *I kind of wish that it was still going the way it was because I would really like to not have to go to the doctor when I need stuff. Just going over the phone, that was pretty rad. (Participant 18; male, 34 years old)* |
|  | OOT contacted individuals less frequently | References to OOT having less frequent contact with participants during COVID-19 | 1 | *…cause there used to be a team that used to come through here like once a week religiously and then I haven't seen them since everything started.*  *(Participant 3; male, 40 years old)* |
| 3.3. Change in Community | Crime increased | References to an increase in crime in participants’ community during COVID-19 | 1 | *… a lot of it's criminal and stuff… there's a lot of people suffering down here, and it's gotten a lot worse, people are stealing a lot more from people that already have nothing. (Participant 13; female, 33 years old)* |
|  | Ghost town | References to participants’ community feeling like a ghost town with empty streets and little foot traffic during COVID-19 | 3 | *It was definitely like a ghost town around.*  *(Participant 5; male, 31 years old)* |
|  | No change specifically downtown | References to the perception that lifestyle changes or impacts due to COVID-19 only occurred outside the downtown core | 2 | *…but in the micro of the downtown east side, the changes are much more subtle. People tend to not socially distance there. They tend to not wash your hands as often, they still share drug paraphernalia and there's, yeah, much less willingness to actually change the routine. (Participant 2; male, 48 years old)* |
|  | Difficulty providing for oneself | References to losing work or having difficulty providing for oneself during COVID-19 | 4 | *Because people are on very limited incomes here and they have their daily hustles. There are things they do to get by during the month and the COVID put a stop to a lot of that for a lot of people. (Participant 2; male, 48 years old)* |
|  | Tension increased in interactions | References to COVID-19 leading to tension in the interactions with one another (e.g. people being more distanced) | 6 | *I find people are a lot more distanced and cold. (Participant 13; female, 33 years old)* |
|  | Overdoses are more important than potential infection | References to participant beliefs that people would not hesitate to help in the event of an overdose despite potential infection of COVID-19 | 3 | *When overdose is happening, who cares about COVID-19? … an overdose is way more lethal than COVID-19. (Participant 11; male, 58 years old)* |
|  | Highlights connection between people | References to COVID-19 having a positive impact on a participant’s community due to highlighting the importance of connections between one another | 2 | *It highlights how important it is to be able to you know, shake a person's hand at the end of the deal like to not be able to like connect with somebody. (Participant 2; male, 48 years old)* |
|  | Difficulty identifying people with masks on | References to participants having a difficulty identifying others during COVID-19 due to people wearing face masks | 2 | *I can maybe recognize a bit of the voice but other than that, the face I can't recognize. (Participant 11; male, 58 years old)* |
| Abbreviations: ED = Emergency Department; OOT = Overdose Outreach Team; OAT = Opioid Agonist Therapy  *https://lifeguarddh.com/  ^1^Pharmaceutical alternatives are substances that can be prescribed to people who use drugs to offer similar therapeutic effects as the toxic drugs they are using but may contain a different dosage form or different salt or ester [Definition from: United States Department of Health and Human Services, Food and Drug Administration, Office of Medical Products and Tobacco, Center for Drug Evaluation and Research, Office of Generic Drugs, Office of Generic Drug Policy. Approved drug products with therapeutic equivalence evaluations. 41st ed. Food and Drug Administration; 2021.] | | | | |
