# Supplementary material for: Experiences of people with opioid use disorder during the COVID-19 pandemic: A qualitative study
Source: PLoS One. 2021 Jul 29;16(7):e0255396. doi: 10.1371/journal.pone.0255396 (PMC8320992; doi:10.1371/journal.pone.0255396)
Supplement: S1 Appendix — Interview guide used to gather participants’ opinions and experiences during COVID-19. (DOCX) [file pone.0255396.s002.docx]

**S1 Appendix. Interview guide.**

Interview guide used to gather participants’ opinions and experiences during COVID-19.

“I am going to start recording now.” **[Start recording by stating interview ID number ___________]**

- 1. To start with, what is you understanding of COVID-19? What have you learned about how to stay safe during the outbreak? How did you learn about COVID-19 and how to keep yourself from getting it?

*[Prompts if needed: Was it from your doctor, friends/family, word on the street, outreach workers, public health officials, social media, other media like TV or radio?]*

- 1. Can you please tell us a little bit about what it’s been like for you during COVID? Did you get sick? Did you get tested? What did you do if you got sick? What did you do to keep yourself from getting infected?
  2. Did you notice a change in your community in the way people interacted with each other? Do you think people were scared to help someone having an overdose? Or scared to help people in other ways? Can you tell us about something you saw or heard that makes you think people either were scared to help or still helped no matter what?
  3. Did COVID-19 affect your ability to get medical care if you needed it? In what way? Were you still able to go to your regular doctor or clinic? Did COVID-19 make it more or less likely that you would go to your doctor? Go to emergency? Call 911? How did it affect your decisions about when and where you needed healthcare?
  4. Did COVID-19 affect your ability to get the social support services you needed? In what way? Did you need a shelter during COVID-19? How did that go?
  5. Did COVID-19 affect your use of drugs? Did you use more/less/different kinds? Did you find you were buying more or less drugs at a time? Did it change your ability to use drugs as safely as possible? For example, difficulty getting safe supplies or getting into an overdose prevention site? Did the social/physical distancing recommendations make you use alone or take other risks you might not usually take? Please explain.
  6. Have you been to an emergency department during the COVID-19 pandemic? Did you notice any changes overall? Did you experience any change in the way you were treated or in the services you were able to receive? Please tell us something about those changes you noticed.
  7. Have you interacted with the Overdose Outreach Team, or other outreach teams during the COVID-19 pandemic? Did you experience any change in the way you were treated or in the services you were able to receive? Please tell us something about those changes you noticed.
  8. Did anyone reach out to you during COVID about getting prescribed a supply of safe opioids? If yes, who reach out to you? Were you able to get a safe supply? Was it helpful?

**“Thank you so much for your willingness to share your thoughts and opinions with us. After we put down into writing the recording of this interview, we may find parts of your responses that may seem unclear to us and we may contact you to make sure we understood what you meant. This concludes interview #____. I will now stop recording.”**
