# Supplementary material for: Experiences of people with opioid use disorder during the COVID-19 pandemic: A qualitative study
Source: PLoS One. 2021 Jul 29;16(7):e0255396. doi: 10.1371/journal.pone.0255396 (PMC8320992; doi:10.1371/journal.pone.0255396)
Supplement: S1 File — Participant transcripts transcribed verbatim from audio recordings with all names or potentially identifying information removed. Unless otherwise specified, “Interviewer” refers to the primary interviewer (LG). (DOCX) [file pone.0255396.s003.docx]

**S1 File. Participant Transcripts.**

Participant transcripts transcribed verbatim from audio recordings with all names or potentially identifying information removed. Unless otherwise specified, “Interviewer” refers to the primary interviewer (LG).

***Participant 1***

Interviewer: So, to start, what is your understanding of COVID-19?

Participant 1: My understanding of COVID-19? What do you mean like the health effects are what?

Interviewer: Like, are you aware of what COVID-19 is and maybe like the symptoms of it?

Participant 1: Well, I'm aware of what COVID-19 is, yes, a flu. Basically.

Interviewer: And what have you learned about how to stay safe during the outbreak?

Participant 1: Stay safe during the outbreak? I don't know. Wear a mask.

Interviewer: Okay, and how did you learn about COVID-19 And how to keep yourself from getting it?

Participant 1: As soon as they announced COVID-19 and I saw the big pandemic that it was creating I thought you know what, I'll be fine. I don't need to worry about this. I mean, I'll wear a mask. I stay away from people, but I'm not staying inside. I'm not staying away from people. I'm just, I'll be fine. I'll keep doing what I'm doing and then I'll be alright.

Interviewer: Okay and when you initially learned about the pandemic, how did you learn? Was it from a doctor or word on the street?

Participant 1: No, it's just news.

Interviewer: Like media? like the radio or?

Participant 1: Yeah, Media. TV.

Interviewer: Can you tell us a little bit, so you said that you've been wearing a mask -

Participant 1: well yeah, if I have to.

Interviewer: Did you get sick?

Participant 1: No

Interviewer: no? And what did you do to keep yourself from getting infected? So you said sometimes you wear a mask -

Participant 1: Nothing.

Interviewer: Did you do anything else?

Participant 1: No.

Interviewer: Okay. Did you notice a change in your community and the way people interacted with each other?

Participant 1: Yeah, I noticed that a lot of people were just buying what the news told them and they thought it was gonna be a major pandemic and kill a bunch of people, which it didn't.

Interviewer: Do you think that people were scared to help someone have been an overdose during this pandemic?

Participant 1: I have no idea.

Interviewer: Okay, or scared to help people in other ways?

Participant 1: I have no idea.

Interviewer: Did COVID-19 affect your ability to get medical care if you needed it?

Participant 1: Yeah, COVID-19 affected my ability to get a lot of things.

Interviewer: Okay and in what ways?

Participant 1: In what ways? Well, I was I was starting a job working for a friend of mine. He's a real estate agent in Kamloops. So and I was going to go up to Kamloops and he had a week of hotels for me up there, because his wife is the manager of a hotel thing or chain up there, not up there but like around Canada, so we had a week of hotels and I was gonna do his whole a whole lot like website end of his business, like SEO and everything to do with that website design. On the day that COVID-19 got announced. So, I had like everything that every last cent to my pocket invested into going up there. And the day that it got announced he cancelled it. I was staying with a friend in Chilliwack gonna go up to Kamloops, he cancelled it. My dad, I got in a fight with him. My dad won’t let me stay with him. I don't talk to my mom. My mom won't let me stay with her. Nothing was open like there was no stores open no. So I was homeless. Rain got on my phone and my laptop. So those were garbage. So I was homeless in, in Chilliwack. And then I came up to Vancouver. And there was nothing out here. So, yeah, it yeah, it affected me big time I couldn't get anything.

Interviewer: Yeah, I'm sorry to hear all of that. That definitely affected you big time.

Participant 1: Oh don’t worry about it, it's already happened.

Interviewer: Did it affect any medical care if you needed it at the time?

Participant 1: Well, yeah, there you can't really couldn't really get anything. I mean, I went into an emergency room and they they told me they gave me some I don't know what they gave me like some band aid or something they told me to leave.

Interviewer: Okay, so did it make you more or less likely to go to the emergency?

Participant 1: Well, I mean, I didn't really have anything wrong with me. So there's not really a reason for me to go like you can't really go just go to emergency room just to hang out.

Interviewer: Right? But maybe to fill, you said sometimes you go to fill prescriptions and everything, did it make you a bit more hesitant to go and to do that or you just had no medical care that you needed to take care of during this time?

Participant 1: I don't know.

Interviewer: Okay. Did it affect your ability to get any social support services? You said that you were - you were homeless for a bit did you did affect any social support services that you needed?

Participant 1: I don't even know what that means.

Interviewer: Like getting help with housing, financial aid and stuff.

Participant 1: I don't even know if there's those were available, so.

Interviewer: did you go to a shelter during COVID-19?

Participant 1: No.

Interviewer: No, okay did-

Participant 1: They don't let you in shelters in COVID-19.

Interviewer: Did COVID-19 affect your use of drugs?

Participant 1: No.

Interviewer: Okay, so it didn't make you use more or less or different kinds?

Participant 1: Well, if I'm homeless on the street, I'm probably going to be using drugs because, yeah.

Interviewer: so did it change your ability to use drugs as safely as possible?

Participant 1: Uh, not - it didn't change my ability to use drugs safely or not, no. Yeah, About the same.

Interviewer: Did the social or physical distancing recommendation make you use alone or take other risks you might not normally have taken while using drugs?

Participant 1: No, not at all. I didn't change anything about being alone or staying away from people. No.

Interviewer: Okay, so you mentioned you did go to the emergency room and they told you, they were very quick with you. So do you believe the experience in the emergency department changed in the way you were treated due to COVID-19?

Participant 1: Um, Not really.

Interviewer: Okay. Have you interacted with the outreach, overdose outreach team or other outreach teams during the COVID-19 pandemic?

Participant 1: Like when you - What's the timeline of the COVID-19 pandemic to you?

Interviewer: I would say maybe like early March

Participant 1: Well, they have, I've interacted with them as much as I have normally.

Interviewer: Okay. Did you experience any change in the way you were treated? Or the services you were able to receive from the outreach team during that time?

Participant 1: No, No.

Interviewer: Okay. And did anyone reach out to during COVID about getting prescribed a supply of safe opioids?

Participant 1: No.

***Participant 2***

Interviewer: So to start, what is your understanding of COVID-19? You know what it is? And you know -

Participant 2: I know what it is, yeah, it is a novel Coronavirus, and we're in a pandemic with it it. Yeah, I know what it is and what it's about, and why we're social distancing, things we're trying to do we're doing to try to mitigate it. And yeah.

Interviewer: What have you learned about how to stay safe during the outbreak?

Participant 2: Basically, don't exchange fluids with anyone, maintain respectful social distance. Wash your hands, don't touch your face, right? Like it's pretty, pretty basic, simple stuff.

Interviewer: How did you learn about COVID-19 and these tips to keep safe?

Participant 2: it's everywhere. I learned because I can read, and I can still hear a little bit. Like it would be it'd be

Interviewer: So was it more word on the street?

Participant 2: Our society was saturated with it. It was saturated with COVID information. I think it would be impossible to not have been aware and of what was happening and know what to do. Yeah, I just don't think you would. Yeah. If you can read and hear then I think you would know because every surface had something on it about it. And yeah, you'd have to know.

Interviewer: So, so the word on the street and ads everywhere posters everywhere. That's kind of how everyone has learnt?

Participant 2: Yeah.

Interviewer: Okay.

Participant 2: radio, television, internet, all media like records and things with everything print, everything was saturated with it, like you couldn't have your head in a deep enough hole to not know what was going on during that time.

Interviewer: Can you tell us a bit about what it was like and what it's been like for you during COVID?

Participant 2: Well, of course there is some anxieties that comes with it, there's a lot of uncertainty in down town east side because people are on very limited incomes here and they have their daily hustles. There are things they do to get by during the month and the COVID put a stop to a lot of that for a lot of people. And so there was a little bit of anxiety of how are people going to make do and manage, right. And so the government stepped up with a couple funding things. Maybe there's one or two out there that people sort of stretched their situation a bit to maybe justify trying to pursue those funding options, but at the end of the day, people managed to get by which was important, but it seems like at this point is people are tired of it, and they just want things to get back to normal and there's less of an acceptance of that this is the new normal, in the downtown Eastside then there was ‘okay, this is what we're gonna have to do for a little whil’e. People are ready for it to get back to where it was before. And they don't like the idea that it's not gonna go to the way it was before.

Interviewer: And how about you? Are you accepting it as the new normal or are anxious to go back?

Participant 2: I'm frustrated by it. I want to know how some things that are near and dear to my heart are gonna work now. I want to know what it's gonna look like. Again, you know, all these answers require imagination. And, you know, we're, we're sort of close to it to to imagine all the fixes yet. You know, it's gonna, that's really going to take some time to see what grows up organically out of the quote unquote new normal. And I don't know we'll have to see

Interviewer: Did you - did you get sick during COVID?

Participant 2: No, I didn't.

Interviewer: Have you gotten tested?

Participant 2: No, I didn't have to.

Interviewer: And what have you done to keep yourself from getting infected?

Participant 2: Well, I wash my hands a lot. I didn't touch my face and I maintained good social distancing. And I just, you know, that's about it.

Interviewer: Okay. Did you notice a change in your community in the way people interacted with each other?

Participant 2: Can you say that again?

Interviewer: did you notice a change in our community in the way people have been interacting with one another?

Participant 2: Yes, in society at large Absolutely. 100% and but in the micro of the downtown east side, the changes are much more subtle. People tend to not socially distance there. They tend to not wash your hands as often, they still share drug paraphernalia and there's yeah, much less willingness to actually change the routine in the [single room occupancies], then there is everywhere else. So it depends on where you fall on the scale, right? Like I spent the majority of my life living, like just any other normal ordinary person, upper middle class. So I tend to to reflect the changes more society in general went through, I kind of went through them, but my peers in my building didn't really go through them as much as I did. So, there is more than one thing happening here.

Interviewer: Do you think people were scared to help someone having an OD?

Participant 2: No, that that, that I didn't see that at all. Actually, I am quite proud of people, no. If someone's overdosing people still stepped up and saves them. Yeah. And we all do that right. We all do that right, Yeah.

Interviewer: Do you think people were scared to help others in other ways?

Participant 2: No, no, well, in the [single room occupancies]? No, no. I don't think people were were more afraid because of COVID in the [single room occupancies] I live in to help, if they're are gonna help they're gonna help. Right?

Interviewer: Okay. So I'm getting like, a no from this, but I'll ask it anyways, just in case you have a comment. Did COVID-19 affect your ability to get medical care if you needed it?

Participant 2: No. no. I saw my doctor, was over the telephone, had an appointment with my GP on the phone, but I was able to get what I needed out of it. So yeah.

Interviewer: So you're still able to access - well you didn't go to your doctor, but you were still able to do an appointment?

Participant 2: Yeah.

Interviewer: Did COVID-19 make it more or less likely that you would make an appointment and see your doctor?

Participant 2: It's more likely I would go see my family doctor because of COVID-19. Yeah, than it was before. Yeah.

Interviewer: Did it make it more or less likely that you would go to the emergency room?

Participant 2: probably less likely.

Interviewer: less likely?

Participant 2: Yeah.

Interviewer: And what would be your reason for?

Participant 2: Just it's hard to get in to.

Interviewer: More people?

Participant 2: No, like less people, just more of a barrier the hospitals became like unwelcoming places.

Interviewer: Okay, did it make you more or less likely to call 911?

Participant 2: Neither, neither, just all likely to call 911 before or after.

Interviewer: And did it affect your decisions about when and where you needed health care?

Participant 2: No, it didn't.

Interviewer: did it affect your ability to get social support services that you may have needed?

Participant 2: No. They're still able to get everything I needed. The people really stepped up for that. It was pretty seamless. Yeah.

Interviewer: And what are some of the social support services that you use that you said were seamless?

Participant 2: just things, interacting with social services was still able to happen. People were still able to get crisis grants and things like that. Like it was still, yeah, business as usual as far as that stuff is concerned.

Interviewer: Okay, and I understand you have housing. So the question is did you need a shelter during COVID-19?

Participant 2: No, I didn't. I didn't use a shelter. I didn't need one either.

Interviewer: Did COVID-19 affect your use of drugs?

Participant 2: No.

Interviewer: You didn't use more or less or different kinds?

Participant 2: Nope.

Interviewer: Did it change your ability to use drugs as safely as possible?

Participant 2: No. It didn't.

Interviewer: Did the social or physical distancing recommendations make you use alone or take other risks you may not have usually taken?

Participant 2: No, I still didn't use alone, like it didn't affect my drug use at all from COVID

Interviewer: Have you been to an emergency department during COVID-19? I know you said you would rather do the doctor. But did you go to the emergency department?

Participant 2: I haven't been to the emergency department since COVID-19. I haven't been in a while. And I'm kind of like, trying to keep that going.

Interviewer: Right. Okay. Have you interacted with the overdose outreach team or other outreach teams during the COVID-19 pandemic?

Participant 2: Yes, I have.

Interviewer: And did you experience any change in the way you were treated or in the services You were able to receive?

Participant 2: No, no. They were still able access what I needed to access. And yeah, it's good.

Interviewer: Did anyone reach out to during COVID about getting prescribed a supply of safe opioids?

Participant 2: Getting a sorry, getting prescribed what?

Interviewer: Did anyone reach out to you about getting prescribed a supply of safe opioids?

Participant 2: Yeah, yep. No, for sure. Yeah. Yeah. Well, I heard about that program. And yeah.

Interviewer: Do you remember who reached out to you?

Participant 2: Do I have what?

Interviewer: Do you remember who it was that reached out to you about getting the safe supply?

Participant 2: I just read I just read on a on a flyer on a on a telephone pole that does safe, safe drugs were available, to talk to your care provider and how to go about getting them so.

Interviewer: And did you go get them?

Participant 2: No, not yet.

***Participant 3***

Interviewer: So, to start, what's your understanding of COVID-19?

Participant 3: I remember it it was first introduced as the Coronavirus, I think. And then I don't know why I haven't seen or had much experience with it. I've just heart lots.

Interviewer: Do you know about the symptoms?

Participant 3: Yes.

Interviewer: And have you - What have you learned about how to keep safe during the outbreak?

Participant 3: What to keep safe?

Interviewer: Yeah. Like what have you learned to help you keep safe during the outbreak?

Participant 3: Just to distance yourself and not, avoid touching other people. Yeah. I think it was good. I thought it was really good to have the social distancing thing I think that's here to stay for good. It should be.

Interviewer: Okay. And how did you learn about COVID-19 and about how to keep safe? Was it like the media? Was it your friends? The word on the street?

Participant 3: Pretty much yeah. family, and the media.

Interviewer: Family and media?

Participant 3: Yeah, media just tried to scare, or it did scare me.

Interviewer: Did you learn a bit from your doctor? You say you go fairly regularly. Did you guys discuss COVID-19?

Participant 3: No, not really. He didn't think it was that big of a deal.

Interviewer: Okay. Can you tell us what it's been like for you during COVID-19?

Participant 3: Yeah, I've pretty much carried on continuing to do what I've been doing, which is nothing but drugs, and you know.

Interviewer: Did you get sick?

Participant 3: No. You know what, I think that I might have already had this like, last year. The year before or something like that, because there's times where I was sick and had the flu like symptoms for like a month.

Interviewer: Okay, but have you gotten sick since that it it hit like everyone here back since March?

Participant 3: Yeah, no, I haven't been sick like that. No, I had food poisoning once.

Interviewer: Have you ever gotten tested for COVID 19?

Participant 3: Yeah, many times.

Interviewer: Many times? Okay. And where were you getting tested?

Participant 3: At the doctor's office and the hospital too, just like quick temperature checks and things like that and then listening to my chest and my breathing or whatever my pulse. Things like that.

Interviewer: Okay, have you gotten the swab test?

Participant 3: No, I never have

Interviewer: No, not that one?

Participant 3: No.

Interviewer: Okay. Did you notice a change in your community in the way people interacted with each other?

Participant 3: Yes.

Interviewer: And what kind of changed?

Participant 3: Definitely slowing traffic down, I mean foot traffic.

Interviewer: Do you think people were scared to help someone having an overdose?

Participant 3: Probably. Yeah. I would have been.

Interviewer: Do you think people were scared to help people in other ways, too?

Participant 3: Yes.

Interviewer: And what makes you think that, that they were scared?

Participant 3: I can just I can only say because I was I experienced those feelings towards others. That I didn't know or that didn't look well. I thought all right and just think the worst, that they were sick or with the Coronavirus or something, with COVID-19.

Interviewer: Okay, did COVID-19 affect your ability to get medical care?

Participant 3: No, I don't think it did.

Interviewer: Were you still able to go to your regular clinic as often as you do?

Participant 3: No, I wasn't.

Interviewer: Okay, so maybe it affected you. You weren't able to go as often as you would like?

Participant 3: No, it was nice to have a little reprieve from the doctor's office. I didn't have to go as regularly.

Interviewer: Okay, okay. So it made you less likely to go to you doctor?

Participant 3: Yeah.

Interviewer: Did it make you more or less likely to go to emergency?

Participant 3: Yes.

Interviewer: And what, more or less?

Participant 3: Less.

Interviewer: Less? did it make you more or less likely to call 911?

Participant 3: No.

Interviewer: Okay. How did it affect your decisions about when and where you needed health care? Did it change how you needed health care and if you went to go get help?

Participant 3: Yeah it did. Yeah.

Interviewer: And how so?

Participant 3: That I would only go if, like maybe like a serious emergency or something very serious.

Interviewer: Okay. Did COVID-19 effect your use of drugs?

Participant 3: I'm going to have to say yes.

Interviewer: Did it make you use more or less or different kinds?

Participant 3: It made me use less, I believe, Yeah, hang on a sec, sorry, yeah it made me use less, and the dope was just shittier.

Interviewer: Did it change your ability to use drugs as safely as possible?

Participant 3: Yes.

Interviewer: And how so?

Participant 3: Cause the overdose prevention sites weren't seeing nearly as many, only half capacity.

Interviewer: Did the social or physical distancing recommendations, make you use alone or take other risks that you may not have normally taken?

Participant 3: Yeah, I would think so. Yeah.

Interviewer: And in what ways? Did it make you use alone where you normally would have used with other people?

Participant 3: Yes.

Interviewer: Okay. Did it make you take risks you might not have usually taken?

Participant 3: Yea, I think of, yes, yeah, you're right. Yeah.

Interviewer: Okay what kind of risks do you think you took?

Participant 3: Using alone and shooting up in my room by myself.

Interviewer: Okay, have you been to an emergency department during COVID-19?

Participant 3: Yes.

Interviewer: Did you notice any changes?

Participant 3: Yeah. I think so.

Interviewer: Did you notice any changes?

Participant 3: Changes in what sorry?

Interviewer: Like in the care they provided?

Participant 3: Yeah, they were definitely more cautious to be around people and the special handling of things and washing of hands.

Interviewer: And did it change- Were you still able to receive all the services you wanted?

Participant 3: Yeah.

Interviewer: Have you interacted with the overdose outreach team or other outreach teams during the COVID-19?

Participant 3: I guess I have. Yeah, I guess it's been going on for quite some time now, hasn't it, it's been a few months. Yeah, yeah.

Interviewer: Did you experience any change in the way you were treated? Or in the services you were able to receive by the outreach team?

Participant 3: Probably.

Interviewer: Can you think of what may have changed?

Participant 3: Yeah, less likely to come by and just knock on the door and say hi, or something cause there used to be a team that used to come through here like once a week religiously and then I haven't seen them since everything started.

Interviewer: Okay. Did anyone reach out to during COVID about getting prescribed a supply of safe opioids?

Participant 3: No.

***Participant 4***

Interviewer: To start with, what is your understanding of COVID-19?

Participant 4: I have a lot of understanding and I don't care.

Interviewer: Okay. Are you aware of that it's a virus? Do you know the symptoms?

Participant 4: Yes.

Interviewer: What have you learned about how to keep safe?

Participant 4: Everything

Interviewer: Everything? And so what what in particular should you do to keep safe?

Participant 4: You leave it your discretion man. And take care of yourself.

Interviewer: And how did you learn about COVID-19?

Participant 4: From the TV and watching news.

Interviewer: Can you tell us about what it's been like for you during COVID-19? Did your life change at all?

Participant 4: No.

Interviewer: No? Did you get sick?

Participant 4: No. I didn't.

Interviewer: Have you gotten tested?

Participant 4: Yes I have.

Interviewer: You have? Okay, And what did you do to -

Participant 4: No, I'm lying. I didn't, no I didn't get sick.

Interviewer: You didn't get sick but you have gotten tested?

Participant 4: Yeah.

Interviewer: Okay. What did you do to keep yourself from getting sick?

Participant 4: I washed my face, my hands, whatever, every day.

Interviewer: Did you notice a change in your community and the way people interacted with each other?

Participant 4: Yeah, people wear masks. And they look like [laughs] never mind. What they look like that's not up to me to say [laughs].

Interviewer: Do you think that people were scared to help someone having an overdose?

Participant 4: Oh, shoot yeah.

Interviewer: And do you think people were scared to help other people in other ways, too?

Participant 4: Yeah.

Interviewer: And was it something you saw or heard about that makes you think this way?

Participant 4: Something I heard about and saw.

Interviewer: Okay, and what did you see or hear about that made you think people were scared?

Participant 4: Oh, this one women, 'help them help them, grab their hands' 'ew i don't know'. She freaked out.

Interviewer: Did COVID-19 affect your ability to get medical care if you needed it?

Participant 4: No.

Interviewer: Were you still able to go to your regular doctor?

Participant 4: Yeah. Well I haven't. My doctor comes to me.

Interviewer: Did COVID-19 make it more or less likely that you would go to the doctor?

Participant 4: Less likely.

Interviewer: Less likely? did it make you more or less likely to call 911?

Participant 4: I haven't had to call them.

Interviewer: Okay. Did it affect your ability to get social support services you may have needed?

Participant 4: No.

Interviewer: Did you need a shelter during COVID-19?

Participant 4: No.

Interviewer: Did COVID-19 effect your use of drugs?

Participant 4: No.

Interviewer: Did you use more or less or different kinds?

Participant 4: Uh, I use a hell of a lot. I don't know.

Interviewer: But during these past few months, do you think you've used more than normal?

Participant 4: Mhmm.

Interviewer: You did? So you think COVID-19 made you use a bit more?

Participant 4: Yep. Actually I was scared for the people.

Interviewer: Did COVID-19 change your ability to use drugs as safely as possible?

Participant 4: No, because I get them off the same dealer.

Interviewer: Did the social or physical distancing recommendations make you use alone or take other risks you may not have normally taken?

Participant 4: No.

Interviewer: Have you been to emergency department during COVID-19?

Participant 4: No.

Interviewer: Have you interacted with the overdose outreach team or other outreach teams during COVID-19?

Participant 4: I haven't OD-ed.

Interviewer: Sorry?

Participant 4: I haven't overdosed.

Interviewer: Okay, have have they contacted you for any other reason during the COVID-19?

Participant 4: No.

Interviewer: No? Did anyone reach out to during COVID about getting prescribed a safe supply of opioids?

Participant 4: No, I always get it off the same person.

***Participant 5***

Interviewer: To start with what is your understanding of the COVID-19 pandemic?

Participant 5: Um, I knew that it was bad.

Interviewer: Okay, and so you're aware it's a virus and you know the symptoms of it?

Participant 5: The shortness of breathing and like coughing.

Interviewer: Okay, what have you learned about how to keep safe during the outbreak?

Participant 5: Um, just to stay away from people.

Interviewer: And how did you learn about COVID-19 And how to keep yourself safe?

Participant 5: Um, I learned about it just from other people.

Interviewer: Other people? Can you tell me what it's been like for you during COVID-19?

Participant 5: It was definitely like a ghost town around.

Interviewer: Did you get sick yourself?

Participant 5: No.

Interviewer: No? Did you get tested?

Participant 5: Yeah.

Interviewer: Yeah. And, and so what did you do to keep yourself from getting infected?

Participant 5: Um well I didn't really changed much I guess.

Interviewer: Okay, did you notice a change in your community in the way people interacted with each other.

Participant 5: For sure.

Interviewer: And in what way?

Participant 5: There were definitely a lot less people around.

Interviewer: Do you think people were scared to help someone having an OD?

Participant 5: I am not too sure.

Interviewer: Not too sure. Do you think people were scared to help people in other ways?

Participant 5: Yeah.

Interviewer: Yeah? And was that something you saw or heard about that makes you think that people were scared?

Participant 5: Something I saw.

Interviewer: Okay. And what did you see?

Participant 5: Just everybody kind of, everyone was really scared.

Interviewer: Okay, and what made you see that they were scared?

Participant 5: Well, they didn't want to be walking around and being near people.

Interviewer: Did COVID-19 affect your ability to get medical care if you needed it?

Participant 5: No.

Interviewer: Were you still able to go to your regular doctor?

Participant 5: I was actually in the hospital when it started.

Interviewer: Okay. Have you needed to go to your regular doctor since then?

Participant 5: No.

Interviewer: Did COVID-19 make you more or less likely to go to the emergency department?

Participant 5: No.

Interviewer: Did it make it more or less likely for you to call 911?

Participant 5: No.

Interviewer: Did it affect your decisions about when and where you needed health care?

Participant 5: No.

Interviewer: Did it affect your ability to get social support services you may have needed?

Participant 5: No.

Interviewer: Did you need a shelter during COVID-19?

Participant 5: Yes.

Interviewer: How did that go?

Participant 5: Well, the outreach team found me a shelter.

Interviewer: Okay. So, you were able to find the shelter you needed?

Participant 5: Yep.

Interviewer: Did COVID-19 affect your use of drugs?

Participant 5: No.

Interviewer: Okay, did you find maybe you were using a bit more or less or different kinds?

Participant 5: I was using more.

Interviewer: Did it change your ability to use drugs as safely as possible?

Participant 5: Well, it shutdown a lot of the overdose sites so yeah.

Interviewer: Okay. Did the social or physical distancing recommendations make you use alone or take other risks you may not have usually taken?

Participant 5: No.

Interviewer: Have you been to an emergency department during COVID-19?

Participant 5: No.

Interviewer: Have you interacted with the overdose outreach team or other outreach teams during COVID-19?

Participant 5: Yeah.

Interviewer: Did you experience any change in the way you were treated or in the services you were able to receive?

Participant 5: Nope.

Interviewer: And did anyone reach out to you during COVID about getting prescribed a supply of opioids?

Participant 5: No.

***Participant 6***

Interviewer: To start with what is your understanding of COVID-19?

Participant 6: Well I understand that it was a pandemic, is a disease that was worldwide and it could possibly be become overwhelming and the death rate could increase if it wasn't taken care of.

Interviewer: What have you learned about how to stay safe during the outbreak?

Participant 6: I learned to wash your hands and you know, wear a mask when you can and try to keep distance from people.

Interviewer: How did you learn about COVID-19? And how to stay safe?

Participant 6: Through pamphlets and radio and television.

Interviewer: Through pamphlets?

Participant 6: Yeah pamphlets, outreach pamphlets.

Interviewer: And where were you getting the pamphlets from?

Participant 6: Addiction, addiction places like you know, addiction, I don't know um harm reduction places, you know.

Interviewer: Can you tell us a bit about what it's been like for you during COVID?

Participant 6: It was shitty.

Interviewer: And why? Why has it been that way for you?

Participant 6: Well, because I had nowhere to go. You couldn't go inside any of the places you weren't able to get into, you know, places to get off the street. And you know, so you if you were homeless, you're on the street. You couldn't get in anywhere. There was no where you could go inside.

Interviewer: Did you get sick?

Participant 6: Yeah, not from COVID-19. But I got sick.

Interviewer: Did you get tested for COVID-19?

Participant 6: No.

Interviewer: And what did you do to keep yourself from getting infected?

Participant 6: I did things that they said I wore masks at times, and I stayed away from people.

Interviewer: Did you notice the change in your community and the way people interacted with each other?

Participant 6: Yeah, big time. Yeah.

Interviewer: Did you, do you think people were scared to help someone having an overdose?

Participant 6: Yeah. Yes there were.

Interviewer: And do you think people were scared to help people in other ways as well.

Participant 6: Yeah, I think generally people were afraid. Yeah.

Interviewer: And was that something you saw or heard about that makes you feel people were scared?

Participant 6: Yeah, the way people reacted and were trying to stay away from other people, and I think they overreacted a bit.

Interviewer: And did COVID-19 affect your ability to get medical care if you needed it?

Participant 6: Yes.

Interviewer: And in what way?

Participant 6: Well, you were limited to the amount of people in the emergency.

Interviewer: So did it make you more or less likely to go to the emergency?

Participant 6: Less likely

Interviewer: Did it make you more or less likely to call 911?

Participant 6: No.

Interviewer: And did it affect your decisions about when and where you needed health care?

Participant 6: No.

Interviewer: Did COVID-19 affect your use of drugs?

Participant 6: Yes, yeah, I used more drugs.

Interviewer: You used more? Did you use different kinds?

Participant 6: No.

Interviewer: Did it change your ability to use drugs as safely as possible?

Participant 6: Yeah, because you couldn't get inside certain places.

Interviewer: Okay, a difficulty getting maybe say supplies or getting into the overdose prevention sites?

Participant 6: Yes.

Interviewer: Did the social or physical distancing recommendations make you use alone or take other risks you may not have usually taken.

Participant 6: Yes.

Interviewer: And can you explain - so did it make you use alone?

Participant 6: It made me use on the street.

Interviewer: Okay. Have you been to an emergency department during the COVID-19 pandemic?

Participant 6: Yes.

Interviewer: Did you notice any changes in the emergency department?

Participant 6: Yes.

Interviewer: And like what?

Participant 6: Less people allowed in at one time.

Interviewer: Did you experience any changes in the way you were treated or in the services you were able to receive?

Participant 6: Not generally, no.

Interviewer: No? Have you interacted with the overdose outreach team or other outreach teams during COVID-19?

Participant 6: Sporadically, yeah.

Interviewer: Did you experience any change in the way you were treated or in the services you were able to receive from the outreach team?

Participant 6: No.

Interviewer: And did anyone reach out to you during COVID about getting prescribed a supply of safe opioids?

Participant 6: I think it was mentioned. Yeah.

Interviewer: Do you remember who reached out to you?

Participant 6: I think it was when I saw [outreach team worker] last time. He mentioned and I thought about it. He just mentioned have I gave, I think last time I saw [outreach team worker] he mentioned have I had ever thought about an alternate, another option.

Interviewer: And did you get a safe supply?

Participant 6: Yeah, I've always gotten it

Interviewer: And was it helpful?

Participant 6: Yes.

***Participant 7***

Interviewer: To start with what is your understanding of COVID-19?

Participant 7: Uh it's a global pandemic, a virus.

Interviewer: What have you learned about how to stay safe during the pandemic?

Participant 7: Protective equipment, mask, lots of washing hands.

Interviewer: And how did you learn about COVID and how to keep safe?

Participant 7: Through [an addiction treatment centre] that I just finished.

Interviewer: And can you tell us a little bit about what it's been like for you during COVID?

Participant 7: Definitely challenging as far as I feel a little isolated from not being able to use various services for my addiction. Like [addiction centre] and even just not having, there was only addiction doctors at the treatment center and a lot of the clinics were closed so it was hard to get anything done as far as getting connected and stuff.

Interviewer: Okay, did you get sick?

Participant 7: No. I was pretty good through it.

Interviewer: Did you get tested?

Participant 7: No.

Interviewer: And what did you do to keep yourself from getting sick

Participant 7: Sanitize things wash my hands lots and I wore masks quite often.

Interviewer: Did you notice a change in your community and the way people interacted with one another?

Participant 7: Yeah, for sure. It created a lot of tension. I think a lot of the times in the treatment center.

Interviewer: Do you think people were scared to help someone having an overdose?

Participant 7: Yeah, definitely. I've seen that. Yeah.

Interviewer: Do you think people were scared to help people in other ways, too?

Participant 7: Yeah.

Interviewer: And is that something you saw yourself or you heard about that makes you think people were scared?

Participant 7: Well, I've seen people like hold their breath and stuff as they're walking by people.

Interviewer: So, you said you have had a little bit trouble getting into clinics. So, did COVID-19 affect your ability to get medical care if you needed it?

Participant 7: Yeah. It was definitely more difficult like to go to get in to see a GP anywhere. Because the treatment center I was at was locked down. So I had to go through all these different steps just to get to see a doctor, I guess.

Interviewer: Were you still able to see a doctor? Eventually?

Participant 7: Eventually, yeah.

Interviewer: Did COVID-19 make it more or less likely that you would go seek a doctor?

Participant 7: I'd say less likely.

Interviewer: Did it make it more or less likely for you to go to the emergency department?

Participant 7: For sure, yeah. I never wanted to be there because of it.

Interviewer: Did it make you more or less likely to call 911.

Participant 7: Yeah, I'd say.

Interviewer: More or less?

Participant 7: Less likely.

Interviewer: And how did it affect your decisions about when and where you needed help?

Participant 7: Like, how, can you repeat that?

Interviewer: How did COVID-19 affect your decisions about when and where you may have needed health care?

Participant 7: Well basically I just didn't want to get infected. So yeah, I just tried to avoid all all the clinics.

Interviewer: Just avoid it?

Participant 7: Yeah.

Interviewer: Did COVID-19 affect your ability to get social support services you may have needed?

Participant 7: Yeah, I just kind of felt that everything, I could never reach anybody and I just I just assumed that everything was closed.

Interviewer: Did you need a shelter during COVID-19?

Participant 7: No.

Interviewer: Did COVID-19 affect your use of drugs?

Participant 7: Um, feeling more isolated. Or you mean like to trigger me or anything is that whatt you mean?

Interviewer: Yeah. Did you use more or less or maybe different kinds?

Participant 7: No, I didn't use it all but definitely triggered use.

Interviewer: Okay. So you said you haven't used at all so we can skip a few of these questions here. Have you been to an emergency department during COVID-19?

Participant 7: No.

Interviewer: Have you interacted with the overdose outreach team or other outreach teams during COVID-19?

Participant 7: Well actually yeah I did go to emerge but I was asking for a mask one day because I didn't have one and the nurse refused to give it to me.

Interviewer: Okay, so do did you experience a change in how you were treated?

Participant 7: Yeah, I don't like, don't feel that she was treating me badly cause I don't think she just thought I was using or anything but she couldn't believe that I was asking her for a mask when she had a ton of them right there. She just, I was just so frustrated, but I guess that’s [Surrey hospital].

Interviewer: Okay, so have you interacted with the overdose outreach team or other outreach teams during COVID-19?

Participant 7: Not much.

Interviewer: Not much. In the times you did, did you experience any change in the way you were treated or the services you were able to receive?

Participant 7: No, everything was good.

Interviewer: And did anyone reach out to you during COVID-19 about getting prescribed a supply of safe opioids?

Participant 7: I was already on it for during COVID.

***Participant 8***

Interviewer: So, to start with, what is your understanding of COVID-19?

Participant 8: Deadly virus, very bad, very contagious.

Interviewer: What have you learned about how to stay safe during the outbreak?

Participant 8: Physical, or like, social distancing, you know.

Interviewer: And how did you learn about COVID-19? And how to keep safe?

Participant 8: Like, everywhere. I heard it on the news first and then, I guess.

Interviewer: Can you tell us a bit what it's been like for you during COVID?

Participant 8: Scary I guess, I'm not sure, unsure, unsettled like I worked right in the middle of it all. Like I mean, in downtown east side, so it's kind of scary there, too.

Interviewer: Did you get sick?

Participant 8: No.

Interviewer: Did you get tested?

Participant 8: No.

Interviewer: And what did you do to keep yourself from getting sick?

Participant 8: Washing my hands, sanitizing, Keeping distance away from people, wearing masks.

Interviewer: Did you notice a change in your community in the way people interacted with each other?

Participant 8: Definitely outside of downtown.

Interviewer: Do you think people were scared to help someone having an overdose?

Participant 8: Yes, I do think that we were all very unsure of what to do, because it kept changing.

Interviewer: Okay, and do you think people were scared to help people in other ways too?

Participant 8: For sure.

Interviewer: Did COVID-19 affect your ability to get medical care if you needed it?

Participant 8: Yeah, cause I just was scared to go to the emergency room.

Interviewer: Okay, were you still able to go to your regular doctor if you needed?

Participant 8: They had like limited hours sometimes and stuff and I actually had my tooth bugging me a lot lately so I've been that's why I've been in the outpatients a lot lately, but like, um, and I had to talk to my dentist over the phone and they couldn't do anything either, so it was kind of all over the phone.

Interviewer: Okay, and so you said you were scared to go to emergency, so you were less likely to go to emergency if you needed medical care?

Participant 8: Yes.

Interviewer: Were you more or less likely to call 911?

Participant 8: Like, if or like, for what?

Interviewer: Like if you ever, if you experienced an emergency maybe it's not applicable, maybe You weren't in a situation that you had to call 911. But if you were, did it make you more or less likely to call 911?

Participant 8: Um like if there's an overdose or something or?

Interviewer: Yes, yeah.

Participant 8: Oh no, I call 911.

Interviewer: Did it affect your ability to get social support services you may have needed?

Participant 8: Nope. Or, yeah. I guess, sorry, in a way. Sorry, yeah.

Interviewer: That's okay. And in what way?

Participant 8: Well, lots of ways, I guess, say like, couldn't meet up with people couldn't go in groups or anything. So you couldn't go anywhere. And isolating. So yeah, definitely just all around affected everything.

Interviewer: Did you need a shelter during COVID-19?

Participant 8: I'm housed right now.

Interviewer: Did COVID-19 affect your use of drugs?

Participant 8: Definitely, it was harder to go out to get it and stuff like that.

Interviewer: So did you find you used more or less or different kinds?

Participant 8: Um, yeah. Definitely different kinds because, like, apparently, like they're having a hard time getting stuff in to make what, how they used to or whatever. So, there's like a lot of different types of dope out. It's weird.

Interviewer: Did you find that you were using more or less?

Participant 8: More, I guess. Because I would buy like, you know what I mean buy bulk, or when I went when I was able to get out or whatever.

Interviewer: Did COVID-19 change your ability to use drugs as safely as possible?

Participant 8: Yeah, for sure, because we had to be by ourselves. We weren't allowed to be around each other. So basically forcing us to use alone.

Interviewer: And did you have difficulty maybe getting safe supplies or getting into an overdose prevention site?

Participant 8: Yeah, definitely. Yeah, that's where I work and I lost work, too. So I had to like quarantine or like self quarantine for a while.

Interviewer: Have you been to an emergency department during COVID-19?

Participant 8: No.

Interviewer: Have you interacted with the overdose outreach team or other outreach teams during COVID-19?

Participant 8: No, I don't think not during COVID

Interviewer: Did anyone reach out to during COVID about getting prescribed a supply of safe opioids?

Participant 8: I was already on.

***Participant 9***

Interviewer: So to start with, what's your understanding of COVID-19?

Participant 9: Um, it's a thing we can't we can't take any risks on man. Like, we have to be really vigilant like being sanitary and our hygiene because if another pandemic happens like this, it's I don't I don't think I don't think humanity could handle a superbug, that superbug like COVID-19 that say we got a cure for COVID 19 but say COVID 19 mutates and is now immune to that cure. I think I, I don't even want to think about what the damage would be man, like it'd be bad.

Interviewer: Right, yes.

Participant 9: But I also think that parents shouldn't baby their kids so much and, and be like, ‘Oh, don't be playing in the mud’. I think back in the 40s, like in 1940s when kids were going outside and getting dirty and weren't receiving as much vaccines and treatment for, like treatment medically, I think they're more resistant to diseases because they were exposed at an early age and their body developed, like, learned to fight it without the use of drugs and medicine. I think I think that if we could somehow do the same thing now, I think it would be really helpful to humanity without having to use drugs so.

Interviewer: Okay. What have you learned about how to stay safe during COVID-19?

Participant 9: Wearing a mask. Yeah, I've got my pocket, like I should be wearing now. But like, the British Columbia like Vancouver Island, they like I was there like, that's like pretty much the safest place in the world right now. As far as COVID-19 is concerned, in my opinion, at least cause I, what, they have the lowest cases, like the lowest amount I'm pretty sure?

Interviewer: I'm not sure I stopped looking at the news because it just got sad.

Participant 9: Yeah, I was told that it was literally like, one of one of the most, one of the safest places on earth right now, like hygienically, I guess.

Interviewer: And how did you learn about COVID-19 and how to keep yourself safe?

Participant 9: Um, I actually learned first heard about it on Facebook. Somebody posted something about it and I was like, ‘What the heck is that like? Coronavirus in China?’ like and then I heard my other buddy was like, ‘Oh, it's a zombie outbreak’. I'm like, ‘oh God’.

Interviewer: Can you tell what it's been like for you during COVID-19?

Participant 9: It's been a been a definite change. I've noticed like, like, [Vancouver street] is like a ghost town. And I used to walk down there at night and like it'd be like everybody would be out at the bar, but it's like, non-existent anymore.

Interviewer: Did you get sick?

Participant 9: No.

Interviewer: Have you got tested?

Participant 9: Yeah.

Interviewer: And what did you do to keep yourself from getting sick?

Participant 9: Um, stayed at home. Followed the advice of that lady, Dr. Bonnie Henry on the news. Yeah, she's awesome.

Interviewer: And did you notice a change in your community in the way people interacted with each other?

Participant 9: Yeah.

Interviewer: Do you think people were scared to help someone having an overdose?

Participant 9: Yeah, I've actually seen that. And I don't think people should, I think people should feel that way but in a way that they should just be reminded to be careful when being close to somebody. I don't think they should be scared to do it. I think they should just be very cautious.

Interviewer: But you saw people scared to interact and help with people?

Participant 9: Hell yeah, like I saw this guy go down and I was the only one helping him because everybody was like ‘Oh I don't want to get Coronavirus’ cause nobody had a mask so yeah.

Interviewer: Did COVID-19 affect your ability to get medical care if you needed it?

Participant 9: Um, not really. Because, like, I have access to a phone, so not for me.

Interviewer: Were you still able to go to regular doctor?

Participant 9: Oh, yeah.

Interviewer: Did COVID-19 make it more or less likely you would go to the doctor?

Participant 9: Yeah, well, no, I didn't have to go as much. And phone visits are awesome. So.

Interviewer: Did it make it more or less likely you would go to emergency?

Participant 9: Um, no.

Interviewer: Did it make it more or less likely that you would call 911?

Participant 9: No.

Interviewer: And did it affect your decisions about when and where you needed health care?

Participant 9: No.

Interviewer: Did it affect your ability to get social services you may have needed?

Participant 9: No, not really.

Interviewer: Did you need to shelter during COVID-19?

Participant 9: Um, yeah, like I do need a place to stay right now. But um, I was told that a lot of the shelters aren't taking anybody. That's what I was told but may have changed now. So.

Interviewer: Did COVID-19 affect your use of drugs?

Participant 9: Yeah. Crystal Meth is very, very, very scarce right now. People are paying like, I think it's $900 an ounce when it was only like $200, like, couple months ago, so, um, and cocaine has doubled in price, yeah it's eighty five thousand dollars a kilo on Vancouver island, so.

Interviewer: So you find you're using much less?

Participant 9: Oh yeah. Well, except for fentanyl, fentanyl has stayed the same. It's still so easy to get.

Interviewer: And did you use any different kinds because of COVID-19?

Participant 9: Well I've smoked more crack now than I have in my whole life because of just like the prices price of meth right now has skyrocketed.

Interviewer: Did COVID-19 change your ability to use drugs as safely as possible?

Participant 9: No.

Interviewer: Did you have any difficulty getting safe supplies?

Participant 9: Yes and no. Yes, because the doctor I just got my script from doesn't support safe supply. And he wouldn't prescribe it to me. He told me I would have to find a doctor that was willing to do it. And so I didn't get my six Dilaudid eights daily as I normally do. Just for a week, but um, it wasn't because of COVID-19 I don't think.

Interviewer: Did these social or physical distancing recommendations, make your use alone or take other risks you may not have normally taken?

Participant 9: Yeah, um, I use the Lifegaurd app. So it's like, it's much easier to use alone and not be worried about not being able to get help. Because if I don't make that one-minute timer, it calls 911, like my phone.

Interviewer: Okay, so COVID-19 did make you use alone. But you made sure you had -

Participant 9: Yeah, it helped me access that, I guess, service more.

Interviewer: Have you been to the emergency department during COVID-19?

Participant 9: No.

Interviewer: Have you interacted with the overdose outreach team or other outreach teams during COVID-19?

Participant 9: Yeah.

Interviewer: And did you experience any change in the way you were treated? Or the services you could be provided?

Participant 9: Well, they way I had to sanitize my hands before I came into the van and these mats and that's about it. And a mask.

Interviewer: That's about it? Did anyone reach out to during COVID about getting prescribed a supply of safe opioids?

Participant 9: Yep. My doctor, um, he actually reached out well, he, he asked me if I, if I was interested in it when I went for my doctor visit because he knows I'm a drug user, right? And I appreciated him telling me about it because it helped me out. It's helping me stay off fentanyl.

Interviewer: Okay, so you were able to get a safe supply and you found it helpful?

Participant 9: Yeah, yeah.

***Participant 10***

Interviewer: So to start with, what's your understanding of COVID-19?

Participant 10: I think I'm pretty like well versed in it. I know that there has been a recent, perhaps outbreak or so. Somewhat. I'm a bit jaded in, in, in what COVID-19 is, I think it's a common cold thing. I think it's a little bit blown out of proportion, to be honest. And I think it needs to be refocused.

Interviewer: And so what have you learned about how to stay safe during the outbreak?

Participant 10: I learned a lot more about like how much people matter and the quality of interaction with people, being able to voice yourself your opinions when you're able to listen to somebody and and connect with that person. I think that's what's missing. And I think that's what it's been highlighted with COVID is, is connected between people. I think it really highlights how important that is.

Interviewer: Have you learned a lot about how to keep yourself from getting infected?

Participant 10: Yeah, I mean, wash your hands and keep yourself safe. Like common cold or or anything, but I think it's more of, of the connection that's really been highlighted and felt and seen that kind of as a positive I think that's a really big thing that a lot of people have been missing is. The focus is to not get infected and oh my god, but really, it's like, what are we missing? And that common connection of what what we're missing and that conversation. It wouldn't have started without it right? So having that as a positive something that is like a huge takeaway and, and, and a really good one. Right?

Interviewer: How did you first learn about COVID?

Participant 10: Just the same as everyone else, the [housing centres] and the original fear of this Corona virus, right like.

Interviewer: Was it from like a doctor or media or-

Participant 10: I guess it was, I guess it was media. Right. And the funny thing about that COVID 19, is it was, it was originally called the Coronavirus, right and originally, if you remember, the President of the United States wanted to build a wall around Mexico, primarily the source of Corona beer. And his inability to do that then turned into you, like, a virus of Corona proportions portions, right. And then it's just kind of grown from from there. Right. And so, again, I know it's a different topic and kind of getting off topic with it, but I am pretty jaded with with the Coronavirus and what is really behind it. I think it's a lot to do with the movement of people and a disconnect between people and a form of control that was tried and maybe has been tried previously, I'm not sure. But I think it just really highlights I think the positive is a highlight of of an importance of connection of people, you know?

Interviewer: And so what's it been like for you during COVID? Did you get sick?

Participant 10: No. I don't actually know anybody who's really gotten sick from COVID. I don't know anybody that knows anybody has gotten sick from COVID.

Interviewer: Have you gotten tested?

Participant 10: Yeah.

Interviewer: And what did you do to keep yourself from getting sick?

Participant 10: Nothing. Not like, not nothing abnormal. That's the thing. It's like anybody that I talk to you, I can, I've asked like, ‘Do you guys know anybody that's been affected with COVID’ and I have never heard anybody say ‘Yeah’.

Interviewer: Did you notice a change in your community and the way people interacted with each other?

Participant 10: Yeah, I think it, one thing that it did do is like, it's a six, six degrees of separation. People have heard that term before, right? Like where it, I could be saying this wrong but within, like we were connected within six people, right? Worldwide.

Interviewer: Yeah I've heard that.

Participant 10: Right and so I think with the six feet of separation required because of the COVID I think the number again, is like important as in terms of like when you're buried six feet deep. I think like the, the like, you know morbid, like that that huge grand like, you know, metaphor like it kind of stands out is it highlights how important it is to be able to you know, shake a person's hand at the end of the deal like to not be able to like connect with somebody. When you and I met today. General any any walk of life or anytime before this Coronavirus, we would have walked up and said ‘Nice to meet you’ and shake the hand and I think that connective, human contact between two humans. It built a trust in a wall of understanding that can't be from an elbow or like, you know what I mean, now it's like this awkward thing of like, oh, COVID, ah ah ah [motions to an elbow handshake]. Right? And it's just something that doesn't need to be. It's just as something that needs to be refocused.

Interviewer: Do you think people were scared to help someone having an overdose?

Participant 10: I think initially, maybe but I don't think it really jumps into play. I think it's really out of out of your mind when you see somebody overdosing. I think that the need for helping the person and the general care for that person's well being supersedes it.

Interviewer: So you don't think you saw a change in the way people helped others?

Participant 10: Maybe, initially, but now? No, I think it's superseded by the actual general care of humans to be.

Interviewer: Did COVID-19 affect your ability to get medical care for you if you needed it?

Participant 10: No.

Interviewer: Were you still able to go to your doctor as frequently as you wanted?

Participant 10: Yeah, yeah, I mean, the only thing it has really done is just slowed everything down. And I think that that's a problem.

Interviewer: Did COVID make it more or less likely you would go to the doctor?

Participant 10: No, I mean people at the end of the day are going to do exactly what people are going to do.

Interviewer: Did it make you more or less likely to go to emergency?

Participant 10: No

Interviewer: Or more or less likely to call 911?

Participant 10: No.

Interviewer: Okay.

Participant 10: I don't think it's changed anybody's life like long term I think maybe initially like panic, but I think after, after the fact like people all over the world, they're gonna maintain the same thing and after a week of doing something just because somebody says you have to, you're gonna find a way around it to get back to the normal like system that works for you. And as soon as like the focus is off and you're out of the lineup waiting to get into whatever retail outlet, you know, you're at home with your family you're not practicing six feet apart, apart. You know what I mean? You're not on the weekend at a house party saying, ‘Oh, sorry, six feet behind you’ before you get a drink at the keg, right

Interviewer: Did COVID affect your ability to get any social support services you may have needed?

Participant 10: No

Interviewer: Did you need a shelter during COVID?

Participant 10: Yeah, I stay in a shelter now.

Interviewer: And how did it go? Was it easy to get a shelter?

Participant 10: Yeah, and it's just the same.

Interviewer: Just the same?

Participant 10: And maybe at first, it was a little bit difficult. I think the one thing that it did do was maybe lift awareness around the importance of maintaining a clean space.

Interviewer: Okay, but did you find it took a little bit longer or about the same to get the-

Participant 10: The same. I don't think anything's really changed. I think it's just like a big like, right?

Interviewer: Did COVID affect your use of drugs?

Participant 10: No.

Interviewer: Did you find you're using more or less?

Participant 10: No, I think everything across the board is the same.

Interviewer: You're not finding you're using different kinds?

Participant 10: No.

Interviewer: Did it change your ability to use drugs as safely as possible?

Participant 10: Did uh. No, I think, I think it may have isolated some people. Right. And I think that's a bad thing. I think it may have but I think people are starting now with summer here. I think people are sick of it and they're just over it and I think it'll it'll eventually just like, like anything that's bullshit. I think it'll just you know, find its way out of the forefront.

Interviewer: You yourself, did you find that the physical distancing made you use alone or take risks that you might not have easily taken?

Participant 10: No, not really.

Interviewer: Have you gone to-

Participant 10: I am sure it has for other, other people like maybe in communities outside of a major city maybe? I think then they'd be more affected by it, like, rural town ships, I guess like, you know.

Interviewer: But you didn't use more alone?

Participant 10: No I think I think where that would come into play is like maybe like Central America or like, you know, places that are not major cities.

Interviewer: Did you- oh sorry. Have you been to an emergency department during COVID?

Participant 10: Yeah.

Interviewer: And did you notice any changes in the way that you were treated or the services you were be able to-

Participant 10: Initially yeah, like, just they maybe developed a system of of the six feet of separation, again, and that's about it.

Interviewer: Did it affect your care?

Participant 10: No, definitely not.

Interviewer: Have you interacted with the overdose outreach team or another outreach team during COVID? rather than this the setting here?

Participant 10: Yeah, Absolutely. Yeah.

Interviewer: And did you experience any changes in the way you were treated or the services you're able to be provided?

Participant 10: No, no. it's actually become like kind of like a unsaid like running joke I think. Like ‘hey COVID’ like fist pump or like, you know, that kind of awkward. Yeah, exactly right. And it's more highlighted the importance of being connected with your friends, your colleagues, your like work relationships and anything else.

Interviewer: And did anyone reach out to you during COVID about getting prescribed a supply of safe opioids?

Participant 10: Nobody that kind of reached out but it is something that um that I have that I have taken advantage of, and I think it's a very positive thing and helped a lot of people in the community. I think it's positive, I think it's a positive thing.

Interviewer: Okay, so no one contacted you but you were able to-

Participant 10: I just heard through fellow people, fellow addicts, fellow in the community. And I think it's a it's a positive thing I think it gives gives like myself or an addict, you know, the ability to, to not feel so stigmatized, right.

Interviewer: So you were able to get a safe supply?

Participant 10: Yeah.

Interviewer: And you found it helpful?

Participant 10: Very much so.

***Participant 11***

Interviewer: So to start with what is your understanding of COVID-19?

Participant 11: Don't cough in my face [laughs]. I leave here I go to work every day. I'm at a construction job. You know, there's maybe 20 guys that are on that job. Nobody wears a mask unless you're chipping concrete and you only wear a mask because you don't want to get concrete dust in your face. But nobody wears a mask because of COVID-19. We have things on there where you're supposed to be like, two feet away, there's signs all over the place. We have a thing where we have to wash your hands every day, or, you know, wash your hands a couple times during the day. People do that. But there are times when you work really close beside somebody. But I know all in all in my job, everybody's really conscious about it. And I don't worry myself that I think we're gonna get sick going to my job site. I mean, you, I look over the fence where I'm at. I'm working right at [Vancouver park], and I look over the fence there is people huddled you know a foot away from each other coughing or doing whatever in their face and I don't see anybody sick. You know, like the whole, all the way up the block there all the way on the on [Vancouver street] there you see people piled on top of each other. You don't see the COVID-19 all down there. You know if anything, it should wipe out all the homeless people cause most of homeless people are right beside each other. They're not paying, doing that two meter part. They don't wear a mask. So I don't really know, honestly, I don't know how, me, honestly, I'm not that worried about it.

Interviewer: Okay. Do you remember where you first learned about COVID 19? Where you first heard about it?

Participant 11: I just looked it up on my phone, I think then there was a bunch of stuff on Google.

Interviewer: So media?

Participant 11: Yeah.

Interviewer: And so did you get sick yourself?

Participant 11: No.

Interviewer: Have you gotten tested?

Participant 11: No.

Interviewer: And so you said you don't wear a mask or anything when you're on work? Is there anything you do to keep yourself from getting sick outside of work?

Participant 11: Well, outside of work, I usually don't go to too many places. Like, really, actually since it started. I put myself at home most of the times and then when I'm here, I don't really hang out with anybody in the building. I just come home through the building, go to my room and that's it. So I'm a bit of a loner that way that I keep to myself. And then on my time off I usually go for a bike ride so biking around past people, and I pass 2 meters away.

Interviewer: Did you notice a change in your community in the way people interacted with each other?

Participant 11: No. It started when I was in this building and it hasn't changed here. People are still the same but I mean, not that many people in the building I really hang out with anyways.

Interviewer: Do you think people were scared to help someone having an overdose?

Participant 11: No, I don't think, like I think about it no, when overdose is happening, who cares about COVID-19? That's way more, an overdose is way more lethal than COVID-19.

Interviewer: Do you think people were scared to help people in other ways, not necessarily an overdose, but maybe if they needed help in some other way?

Participant 11: Helping in what other ways, like?

Interviewer: I don't know maybe someone fell down? And they were scared to go help them?

Participant 11: I actually honestly haven't seen any that. No, if somebody somebody needs help, COVID-19 to me, I don't think I've actually witnessed that where somebody has refused to help someone.

Interviewer: Did COVID-19 affect your ability to get medical care if you needed it?

Participant 11: No.

Interviewer: And were you still able to go to regular your regular doctor?

Participant 11: Yeah, when I when I, COVID19, I was starting to see the [Vancouver addiction clinic]. So I seen the doctor up there and then COVID-19 got worse and worse and worse. And then I stopped going to see the doctor. All I had to do was call him. And so we went through probably about two months of that where I just called him and I got my prescription and that was that.

Interviewer: Okay, so you didn't physically see but you're still able to get as much as the services as you need?

Participant 11: I was still able to get my, my prescription just carried on and on, and everything was just fine. And COVID-19 never stopped it anyways.

Interviewer: Did COVID-19 make it more or less likely that you might go to the doctor?

Participant 11: No.

Interviewer: More or less likely that you would go to the emergency?

Participant 11: No.

Interviewer: And more or less likely that you would call 911?

Participant 11: No.

Interviewer: Okay, so I guess it didn't affect your decisions about when and where for health care at all?

Participant 11: No, no no.

Interviewer: Did COVID-19 affect your ability to get social support services you may have needed?

Participant 11: Nope.

Interviewer: And the question is, did you need a shelter during COVID-19? I'm gonna say no, because you said you've been in here for over a year?

Participant 11: Yup. I've been here.

Interviewer: And did COVID affect your use of drugs? So you said you've been on Suboxone daily since-

Participant 11: Since Christmas. So COVID was at Christmas, wasn't it?

Interviewer: So have you not used since, since Christmas?

Participant 11: I used probably twice since Christmas, just a small amount, but I have used.

Interviewer: And do you think COVID-19 was the reason for it?

Participant 11: No.

Interviewer: So COVID-19 didn't make you more or less likely to use drugs?

Participant 11: No.

Interviewer: We can skip all these questions then. And have you been to an emergency department during COVID?

Participant 11: Nope.

Interviewer: And have you interacted with the overdose outreach team or another outreach team during COVID?

Participant 11: Yeah, yeah.

Interviewer: Did you experience any change in the way you were treated? Or maybe the services you're able to be provided?

Participant 11: No, other than people show up with masks on their faces [laughs]. They're hard to recognize. I can maybe recognize a bit of the voice but other than that, the face I can't recognize.

Interviewer: And so I know you said you've been on Suboxone, but did anyone reach out to you during COVID about getting prescribed a supply of safe opioids?

Participant 11: Say that again.

Interviewer: Did anyone reach out to you during COVID-19 about getting prescribed a supply of safe opiates?

Participant 11: No. Is, is there such a thing getting supply of safe opiates like what?

Interviewer: I think they came out with a supply of like of safe opioids in the terms of manufactured and I think they came up with this the supply-

Participant 11: No one has ever contacted me about that, but I heard about it. Yeah, like I know, I remember the name of it, but I didn't know what you were talking about.

Interviewer: But no one contacted you during COVID?

Participant 11: Nope, no no.

***Participant 12***

Interviewer: So to start with what's your understanding of COVID-19?

Participant 12: Um it is a kind of like a SARS. It's like SARS I think, it's it's got a high, like, contagious, like passing it rate, like very high, but a low, like whatever you call it, mortality rate, right. So it's easy to pass and and infect it but you, a lot of people wouldn't really, if you have it and your immune systems okay, notice it. I don't know, I have no idea because I don't know if like anybody I know has had it right. What I do know is that like, one thing is like if you have respiratory issues that you will be more susceptible to like it being worse for you, right? I watched actually a thing on it and I know everything about it but it was like in such a doctor like, I couldn't, I don't know what the heck.

Interviewer: It was too technical?

Participant 12: Yeah way too for me like, you know, I could I, yeah there's terms like are coming right out of the medical dictionary like I don't know but I mean, I have a pretty good understanding of it.

Interviewer: And what have you learned about how to stay safe?

Participant 12: Well. I have, well, there's all sorts of different things that people think is what you need to do. I know that it is from like the droplets out of your mouth. So the sanitizer is a big thing for things that you touch, covering your mouth when you cough, washing your hands is important. Like yeah, that's basically the best thing to do is washing your hands. I think that like the not being able to go anywhere, is great, but I don't know if that is still a thing. if we are able to leave the country or not.

Interviewer: I think we can travel within Canada. But I think the border is still closed.

Participant 12: Yeah. I think it's like that's the best thing because like honestly, it's like it's like they said there's going to be second waves and third waves because they have no, they have like an idea of what you can do like rheumatoid arthritis like medication I think might help it or something because it's makes your immune system, I don't know what it is, but they don't really have like a cure for it.

Interviewer: Do you remember how you first learned about COVID-19?

Participant 12: Yeah, I was in the hospital actually, January for my like, it was the time I was, the survey time. It was like the middle of the night at this point though, and I saw that there was on the news in the hospital there's like first outbreak of this new thing in Wuhan and yeah it was pretty scary, like, it was coming up to be Chinese New year and yeah, that was the first time I heard about it or like learned about it. It was like beginning in January and then and then in like February at the end of February, everything started to get really, really scary. Like really quick.

Interviewer: Can you tell us what it's been like for you during COVID?

Participant 12: Oh, it has been interesting. I think there was like, I don't know, it was like we went from one way to the to the next like, I don't know, it's I think, it's just like a almost a little bit too much of a scare tactic. But at the same time, it is very real. Like I know it's real. It's not, it's not like it's not a real thing. But I don't know, then there's conspiracy theories on it. And there's all sorts of things but I know that during the pandemic, when everything was shut down, that was a terrible. That was terrifying. That scared me to see your whole entire city just like go dark. I was alone at the time, my boyfriend was in jail. So like, it was really scary. I would not want to experience that again.

Interviewer: Did you get sick?

Participant 12: No, I, not like I could tell. But I think I would know if I did because I have, like, respiratory issues when I have like a panic attack I can't, yeah, I would know if I got sick. I would like to think.

Interviewer: And have you gotten tested?

Participant 12: I haven't. That's one thing I think is they need to have more like testings, or regular test sites or something. I have never even seen one or been offered one.

Interviewer: And what did you do to keep yourself from getting infected?

Participant 12: I stayed, well, I don't know, didn't really do a lot. I mean, I, my life didn't. I mean, it changed in the way that like everything was closed, but like the only thing that was easily accessible was drugs. So it was like it was kind of backwards for me. I didn't really do, I stayed home when I could. I would buy bigger amounts of drugs I guess to be able to stay home longer and not have to go out and get them, you know. Because there's like a huge increase in people on the street because of the not allowing them in the [single room occupancies] and visitors and all that like it just made like the streets be flooded with death and down here, not everywhere but just like, so I tried to stay away from downtime east side the best I could.

Interviewer: Did you notice a change in your community and the way people interacted with each other?

Participant 12: Yes. Yeah, definitely.

Interviewer: And what kind of change?

Participant 12: Like less like touching and like yeah more closed off and I don't know.

Interviewer: Do you think people were scared to help someone having an overdose?

Participant 12: Probably. Yeah, probably, do I think that do I feel that people are, yes, definitely. I mean, like, I think that people would be more worried of like a homeless person on the street living on the street than somebody who doesn't or that isn't an addict or anything like that. I think anybody would be more worried to help out somebody who's, well looking out for their own personal health and safety.

Interviewer: Do you think people would, are scared to help people in other ways, too, not necessarily an overdose but -

Participant 12: Yeah.

Interviewer: And is this something you saw or heard about or feelings maybe that you've had yourself? What makes you think that way?

Participant 12: Yeah I just think that way because yeah, like I said, like I myself wouldn't want to personally. So if I as an addict feel that way about other addicts then I could only imagine what other people would feel and think so.

Interviewer: Did COVID-19 affect your ability to get medical care?

Participant 12: A little bit. I was still able to get a prescription if I wanted to from my family doctor from my methadone clinic, but I didn't want to go to the emergency at all like at that time. So the first time I went it was just like a week ago or four or five days ago, because I didn't know what was gonna happen if I went in there. I thought they were gonna think that I was sick or that is why I was coming there so I didn't go.

Interviewer: And so you said you don't have a regular doctor right now, but you can-

Participant 12: Yeah I have a clinic I go to, a regular clinic, [Vancouver clinic], but I just like have a few different doctors I've seen there.

Interviewer: And so you said it kind of affected your medical care?

Participant 12: It did yeah, it definitely did. Yes, for sure.

Interviewer: What kind of way?

Participant 12: Because like well everything was closed, so we couldn't go into the doctor. I think I just got out of detox, yeah I just got out of detox at the time and there was no, yeah, and that's why I ended up going off my methadone because like the pharmacy was closed and the doctor's office was closed and you can still get a prescription if you wanted but then you had to take it to a different pharmacy like Shoppers and only during certain hours, you know, so yeah it was hard.

Interviewer: So if the doctor was open, did COVID make it more or less likely you would go to the doctor?

Participant 12: Um. No, no. uh. Yes, yes. Sorry. It does.

Interviewer: Make you more or less?

Participant 12: Mo- Less likely, yeah.

Interviewer: Less likely? And it sounded like it made you less likely to go to the emergency?

Participant 12: Yes.

Interviewer: Did it make you more or less likely to call 911?

Participant 12: Um.

Interviewer: Or no changes is also an option?

Participant 12: No, no changes. I actually did call 911 once when I was, during the thing. Yeah.

Interviewer: Okay. So, no changes?

Participant 12: No, no, no change. I called them, I was honest. I told them they should wear hazmat suit. And they were like terrified when they got to my house, but then they left right away because my friend was like, I don't need it and they're like 'okay goodbye’. Normally they're more pushy about it, but this time they were like, ‘okay goodbye’, because I said like you may want to wear a hazmat suit just because of my room though not because there's anybody sick there but they probably took it like that.

Interviewer: Did it affect- Did COVID-19 affect your ability to get social support services you may have needed?

Participant 12: No, no.

Interviewer: Did you need a shelter during COVID?

Participant 12: No.

Interviewer: And so you mentioned that you've found yourself buying more drugs that at one time-

Participant 12: Yeah.

Interviewer: Did it affect, did COVID-19 affect your use of the drugs? Did you find you were using more or less?

Participant 12: Yeah, a lot. A lot more. Definitely.

Interviewer: Were you using different kinds?

Participant 12: I started, that's when I started to use the, like benzos mixed in with the fentanyl.

Interviewer: During COVID?

Participant 12: Yep. During the COVID I started using it more, a lot more and I don't know why if it had anything to do with COVID or if it's because my partner was in jail or if it was just because I had a lot of people at my house and it was really stressful because like, I was trying to do the right thing and help people from getting off the street. But at the same time, I'm putting myself in danger as well by doing that, so I, like it was a stressful time because I'm too nice of a person to like, turn them away at this time and then they all, so a bunch of, you know, like, it was stressful. Yeah, definitely stressful because I'm a more helpful addict and I have a little bit more stability than other people. So I'm always trying to help but I can't help anybody, I can barely help myself, right. So it was, put myself in some pretty hairy predicaments. But whatever.

Interviewer: Did COVID change your ability to use drugs as safely as possible?

Participant 12: Yeah, yeah. Yeah. Are you asking if the sites are different, like the the injection safe injection sites are different now? They definitely are yeah. I don't go to them anymore now because of it.

Interviewer: And when you say they're different, what do you mean they're-

Participant 12: Well like just how they only allow like a certain amount of people in. There's no waiting room inside. I'm not saying that everything was perfect the way it was before, but it was definitely more likely for me to go in and use that place that service then, then is now. I don't even bother.

Interviewer: Because the wait time would be too long?

Participant 12: Yeah, the wait times too long. And yes, just totally yeah.

Interviewer: Did these social or physical distancing recommendations make you use alone or take other risks?

Participant 12: No. Not that I can think of. I mean.

Interviewer: So the biggest change to your safety is you didn't use those, like overdose prevention sites?

Participant 12: Yeah, I didn't use the, and I would end up be using in the alley.

Interviewer: Did it make you use more alone in the alley or would you always have someone with you?

Participant 12: I always have somebody with me. Ever since I locked myself in the bathroom alone to use and like I usually am pretty, I try to have somebody with me or I try to be in a busy spot that I'm not gonna be overlooked.

Interviewer: Have you, yes you said you've been to the emergency room during COVID-19? You went just this just recently?

Participant 12: Just recently, Yeah.

Interviewer: And did you notice any changes?

Participant 12: Yeah, it's a whole different area.

Interviewer: Did you experience any changes in the way you were treated? Or the services you were able to receive?

Participant 12: It was a little bit different this time, Yeah at [Vancouver hospital]. The doctor was a little bit more judgmental, like I said. And and not, not the same overall vibe that I get from this place.

Interviewer: So a little bit less comfortable this time or-

Participant 12: Yeah, definitely at [Vancouver hospital]. It, definitely a little bit more uncomfortable.

Interviewer: And were you still able to receive all the services that you wanted? Or did you notice a change in that too?

Participant 12: No, I mean I was able to receive the services, yeah, but I I don't know. Yeah, there's there's not all of them, like, actually, that's not true. I think they were actually more available because they wanted to get people off the street drugs more. So I feel like they were starting to like produce more like pharmaceutical like the therapy like the Dilaudids and everything I think it was actually got easier, like better. Now that I think about it.

Interviewer: Okay, and have you interacted with the overdose outreach team or other outreach teams during COVID-19?

Participant 12: Well, I didn't but my roommate at one time, she's not there anymore, but she did have like some workers come by that were, yeah, she always had all sorts of like services like people like cause she was she was doing like a statement again, like the police and sexual abuse. So she always, all kinds of people come by and help us but I don't know if it was an overdose outreach thing.

Interviewer: Okay, and no one personally for you?

Participant 12: No, not for me. Yeah. But I mean, afterwards it was personal for me. I met them. You know what I mean.

Interviewer: You were there-

Participant 12: Yeah, for sure.

Interviewer: And even if it wasn't an outreach team, did you find that the way they treated you or the services you were able to get from them were any different because of COVID?

Participant 12: No.

Interviewer: Did anyone reach out to you during COVID about getting prescribed a supply of safe opioids?

Participant 12: Yes.

Interviewer: Yeah? Do you remember who reached out to you?

Participant 12: My partner.

Interviewer: Your partner?

Participant 12: Yeah. Because he was starting to get out and he figures oh because like if he gets out to me using he's going to start using too And that's just a fact. So he used one and told me ‘you go and get it’, but it was just too little too late I supposed so now I got to figure it out.

Interviewer: Do you remember where he heard about it?

Participant 12: I think on TV, on the news or something, anywhere in the paper, I have no idea, somebody else in there.

Interviewer: And you said you weren't able to get a supply, you didn't get one yourself?

Participant 12: Nope.

***Participant 13***

Interviewer: So what is your understanding of COVID-19?

Participant 13: That it was a disease that was came from China, from bats or something like that, and was, was either that or it was made in a lab and they planned it and I think I'm leaning more towards that it was planned to for population control.

Interviewer: And what have you learned about how to stay safe during the outbreak?

Participant 13: To wear masks and distance ourselves from others, safe distancing, isolation, staying away from people, staying inside more that kind of stuff. Washing your hands a lot, sanitizer and yup that kind of thing.

Interviewer: Okay, and where did you first learn about COVID-19 and how to keep yourself safe?

Participant 13: Just like, everywhere like on the signs and stuff on the on my building and like clinics and that type of thing.

Interviewer: So like, would you say it was from your doctor or word on the street more thing or from friends-

Participant 13: From the media like advertisements, friends and from TV.

Interviewer: Okay. And can you tell us what it's been like for you during COVID?

Participant 13: The beginning was frustrating. And now I feel like I don't really think about it. But I know that it's happening still. And I don't know how I feel about it. I feel like I'm left in the dark about it, because I don't have a cell phone. I have no access to internet at the moment. So, I really don't know what's going on. And I don't know, I'd kind of rather have it that way, in a sense, but at the same time, I don't know. I don't I don't know what to think about it. I don't think it's a good thing. Obviously, it's pretty scary, but I just try to I don't know. Just follow what they suggested and carry on with life, I guess.

Interviewer: And did you get sick?

Participant 13: No.

Interviewer: And did you get tested?

Participant 13: No.

Interviewer: And what did you do yourself to keep from getting infected?

Participant 13: Nothing different, masks now and then, maybe a little extra sanitizer and I've always washed my hands a lot. So.

Interviewer: Did you notice a change in your community in the way people interacted with each other?

Participant 13: Yeah, I find people are a lot more distanced and cold.

Interviewer: Do you think people were scared to help someone having an overdose?

Participant 13: Oh, for sure. They're scared to come around us at all. They think we're dirty.

Interviewer: And do you think people were scared to help people just in other ways too, not necessarily an overdose but something else?

Participant 13: Yeah, they're scared to donate now. And they're scared to buy things from us. Like, I don't know, the way that some people support themselves, they, you know, sell things and that whole industry is collapsed basically, for those people. It's just it's kind of a mess down here. You know, like, I know a lot of it's criminal and stuff, but it's just there's a lot of people suffering down here, and it's gotten a lot worse, people are stealing a lot more from people that already have nothing, you know, and it's pretty shitty.

Interviewer: Did COVID-19 affect your ability to get medical care if you needed it?

Participant 13: Not really, no.

Interviewer: Were you still able to go to your regular doctor or clinic if you needed to go?

Participant 13: Yeah.

Interviewer: Did COVID-19 make it more or less likely that you would go to the doctor?

Participant 13: Nope.

Interviewer: Did it make it more or less likely you would go to emergency?

Participant 13: Nope.

Interviewer: Did it make it more or less likely that you would call? 911?

Participant 13: No.

Interviewer: Okay. Did it affect your ability to get social support services you may have needed?

Participant 13: I believe so. Probably, it slowed it down.

Interviewer: Okay. So slowed it down. any other, were you, like denied any services you were looking for? Or is there any other way-

Participant 13: Recovery houses, treatment, housing, that type of stuff. You know, it got shut down for a while nobody was doing anything. Right. So that really sucked. And yeah, like it slowed that whole process down because they wanted to figure out a way of, you know, making sure everybody was not sick, going into treatments and whatnot. So for new for new patients, new residents, so. Yeah, I opted out I didn't end up going to recovery, now I don't know if I want to go. But I mean so how.

Interviewer: Did you need a shelter during COVID?

Participant 13: Pardon me?

Interviewer: Did you need a shelter during COVID-19?

Participant 13: I wouldn't go to a shelter right now during COVID.

Interviewer: Oh you are already-

Participant 13: I have housing I just didn't, I wanted to go to a treatment center. My housing, people use in my housing.

Interviewer: Okay, but you didn't go seek a shelter during COVID?

Participant 13: No, because I got housing, I have housing.

Interviewer: Perfect. Did COVID-19 affect your use of drugs?

Participant 13: Um, should have, maybe I use more of my, I didn't share supplies as much like hooters or smokes.

Interviewer: Okay, did you find you were using more or less drugs?

Participant 13: No difference.

Interviewer: No difference in how much you were using? Okay. Did you find you were using different kinds because of COVID?

Participant 13: Actually less speed because it screwed up the borders.

Interviewer: Okay, so less of some particular kinds?

Participant 13: Less access to, less access to the crystal meth and speed because of all of it. So now people have to pay more, and we can't afford it. So, going with out speed, which has been hard.

Interviewer: And did you find that you were buying more or less at a time?

Participant 13: I'm buying way less.

Interviewer: Did it change, did COVID change your ability to use drugs as safely as possible?

Participant 13: Yeah, because the safe injection sites were closed.

Interviewer: And did the social or physical distancing recommendations make you use alone or take other risks you may not usually have taken?

Participant 13: No.

Interviewer: Have you been to an emergency department during COVID-19?

Participant 13: Yeah.

Interviewer: And did you notice any changes in the emergency?

Participant 13: They're very strict about washing hands and stuff at the front door.

Interviewer: Did you experience any change in the way you were treated or the services you were able to receive?

Participant 13: I'm not sure I didn't really look into that many services at the time.

Interviewer: Okay, but when you went to the emergency, apart from like, the hand washing, did you notice any differences in how you were treated and the care they gave you?

Participant 13: Just a lot more, I don't know. They just I guess they just only more paid attention to the most serious stuff like whatever seems less important was kind of just you know, put to the side.

Interviewer: Okay. Have you interacted with the overdose outreach team or other outreach teams during COVID?

Participant 13: No.

Interviewer: And did anyone reach out to during COVID about getting prescribed to supply of safe opioids?

Participant 13: I heard about it through my clinic.

Interviewer: Okay, so no one reached out to you, but you heard about it elsewhere.

Participant 13: Yeah. It was word of mouth or something like that.

Interviewer: Okay. And did you go get a safe supply?

Participant 13: Yeah, but it doesn't make a difference because I'm not a pill person, it's not the same drugs.

Interviewer: Oh, so it wasn't helpful for you?

Participant 13: No.

[*Note*: This last quote from Participant 13 came from an interview regarding experiences in emergency departments. This interview was part of the same research project and the COVID-19 interview was done immediately afterwards. This quote has been included in this transcript and included with the COVID-19 data because of its relevance to COVID-19.]

Interviewer: …What are some of the bad experiences [in the emergency department] that are most prominent in your mind?

Participant 13: Okay, probably the most prominent one was, first of all, for with during COVID not being allowed to go in with my my partner, you know, when he overdosed. Like he he was brought back at the scene, and he's terrified of going to hospitals because he lost his previous like wife and daughter in the hospital. You know, like 10 years ago, so when he came out of his overdose at the scene, they didn't, I wasn't allowed to go to the hospital with him. They wanted to check up on him because he's got cancer and some other serious health things. You know, he's scared he's gonna pass away if he go, when he goes in, right. So I find that really difficult and hard to deal with, not being able to go to the emergency room like as a visitor even like, during COVID that wa- I know there's not much you can do about it, but it's just, yeah, I was, I don't know if they can somehow, they could have done it a little bit differently …

***Participant 14***

Participant 14: And the COVID questions wouldn't be um, I don't, I haven't had it. I don't have any friends who have been diagnosed with it. Not to my knowledge and also yeah, I just, I've been self isolating. So I don't know very much about it. Other than what I've been told by you know, by the radio or by friends by hearsay, by the posters everywhere and other than that, I haven't seen anybody who's sick, I haven't really come in contact with it. Honestly, the the the more I know about it, the less time wanting to, you know what I mean and the less prepared I'll be if I yeah, I don't know I like to at least compare theories. I like to have an understanding but more and more you know, if somebody has a first hand experience, so yeah. I was, I was in the hospital recently and yeah. They tried to put me in the, in the COVID, with the in the in the area where the where the people with who had been diagnosed with it, in that area. And I was extremely dumbfounded. I actually was really, really upset because I had had not been diagnosed with it. And when they tested me, I the results came back negative. And so I was extremely, extremely upset. And I told them this if this is an aggressive virus, and you're going to put me there, like, I'm vulnerable, and you're putting, you're putting me at stake, right, like so that's that's honestly, that's all I'd like to say about it. At this point. I don't really I don't really think I'd like to go too deeply into.

Interviewer: What have you learned about staying safe?

Participant 14: About staying safe?

Interviewer: Yeah.

Participant 14: Well, honestly, I think that the methods that we're using, now at this point in time are not as, we're using faulty methods. We're going based on assumptions. And I think that the best method to use would be just to, I mean isolation is important, but it depends on where you live, right? If we live in buildings like this the air circulation, the air is circulated. It's a, it's recycled air. And now if you think about it, in terms of transit, it's recycled air. If you think of it in terms of people wearing masks, you're you're only, like you need a two-way breathing mask. You need a two-way airflow. It depends on what kind of, it depends on what kind of tracheal system you have. It depends on what kind of health you you have, right, what kind of what kind of, the function of your organs it depends on, everything. It's all personalized. It's all singularly we all have unique health, right. I think that social distancing, it's a personal thing. It's something that shouldn't be, it shouldn't be like, linear. I think that people should know what personal, what social distance is just based on how society is right. Some people they require, say somebody to be at their side, right. Some people require that and society, you know, I've heard that there's been some negative some negativity towards, you know, certain, Bbasically, the legal legal aspects of militant force being used and people being incarcerated due to not respecting the boundaries or the or the laws thats placed.

Interviewer: Have you-

Participant 14: I think- I think it's ridiculous.

Interviewer: Have you done anything yourself to keep yourself from getting infected?

Participant 14: Yeah, I've been, I've been doing everything that I can. I keep myself, I try to keep as healthy as possible.

Interviewer: And do you remember how you first learned about COVID?

Participant 14: Probably word of mouth.

Interviewer: Word of mouth? Okay. And did you notice a change in your community and the way people interacted with each other?

Participant 14: I just remember a lot of panic. A lot of panic and distress.

Interviewer: Do you think people were scared to help someone having an overdose because of COVID?

Participant 14: Mhmm.

Interviewer: Do you think people were scared-

Participant 14: I think that generally people are afraid to help people having an overdose. But you know, and honestly, people were all too eager to help somebody if they had Narcan. But if we have a more more depth to understanding of the way that the intricacies of of life and death and how people need to be treated and how the world needs to respond and in order to, in order to maintain respectfulness and awareness of who we are and how we are, you know, coming to be. I think that we have to have a better understanding of how to how to go about this instead of pumping each other with Narcan. We need to have a more thoughtful, more aware adjustments to how to bring somebody back, right.

Interviewer: And just the very first part of your answer kind of got muffled, do you think people were scared to help people?

Participant 14: I think people should be afraid to help people if they don't know how to help people.

Interviewer: Okay, but do you think people were scared because of COVID?

Participant 14: No, I think people are afraid because it's there's a social, there's a social, we're in a state of a of a, there's an extremely, extremely, I can't I can't really explain it. But if I could explain it to you, it would be something very vague. Like we are in shit. We're in shit and turmoil and the only way to get out of it is to learn and knowledge is the only way that we can deal. It's the only way.

Interviewer: And did COVID-19 affect your ability to get medical care if you needed it?

Participant 14: No

Interviewer: Were you still able to go to your regular doctor if you needed?

Participant 14: Yeah.

Interviewer: Did COVID make it more or less likely that you would go to your doctor?

Participant 14: No.

Interviewer: Did it make it more or less likely you'd go to the emergency?

Participant 14: Yes, it did.

Interviewer: More or less?

Participant 14: I'm answering that one just generally because I think that for me, it's the answer would be based on how I feel about in relativity to everybody. Because that's the, that's where people would go right now. That’s, that's exactly how people are going to get carpooled or caravanned into

Interviewer: Okay, so it sounds like it would make you less likely to go to emergency?

Participant 14: No, it actually made me more likely. That, the first time I heard about COVID actually had just come from the hospital.

Interviewer: And did COVID make it more or less likely that you would call 911?

Participant 14: I don't call 911.

Interviewer: Did it affect your ability to get social support services you may have needed?

Participant 14: Actually I think it's made it, it's made it like a ridiculously like a at a false. It's, it's made people in the society abuse, abuse social. So the social system in place the welfare and the social assistance, it's abusive.

Interviewer: Okay, but you personally, did you need any services? And did it affect your ability to get them?

Participant 14: Hmm, no, I find that like, over the years I've ever I've practiced, I'm, I'm trying to come to a point in my life where I can, I can support myself. I feel that that's a that I'm not disabled and that I feel like I deserve to give that to myself, you know, to be able to support myself is it's actually a goal of mine is something that I strive for.

Interviewer: Did you need a shelter during COVID?

Participant 14: No. I had housing.

Interviewer: Did COVID-19 effect your use of drugs?

Participant 14: No.

Interviewer: Did you find you were using more or less?

Participant 14: No

Interviewer: Or different kinds?

Participant 14: No

Interviewer: Did you find you are buying more or less at a time?

Participant 14: I found that everybody around me getting their money, their support, acquired a place to stay or somebody to spend time with or what have you. I think that if we, if self isolation is something that we need to do then helping people get housed is really important.

Interviewer: Absolutely. Did COVID change your ability to use drugs as safely as possible?

Participant 14: Yes.

Interviewer: It did?

Participant 14: Yeah.

Interviewer: Did these social or physical distancing recommendations, make you use alone or take other risks?

Participant 14: Most likely.

Interviewer: And did you find that you were buying more or less drugs at a time?

Participant 14: Um, I actually haven't been paying too much attention.

Interviewer: Sorry, can you repeat that?

Participant 14: I haven’t been paying too much attention.

Interviewer: Oh okay. Not noticeably? Sounds good. Have you been to an emergency department during COVID-19?

Participant 14: An emergency department, elaborate?

Secondary interviewer (JH): Like at a hospital. Have you been to an emergency department at [Vancouver hospitals]?

Interviewer: During like the last six months or so?

Participant 14: Sorry I'm thinking really hard. I'm not too sure but I just have a recollection of something that had happened and something really, uh actually requires medical attention.

Interviewer: Okay, you don't think you've been to the emergency department during COVID?

Secondary interviewer (JH): Do you want to take another little break, maybe?

Participant 14: How many more do we have?

Secondary interviewer (JH): There's about I'd say 10 minutes left, maybe, maybe about. Maybe we'll take a little break. I'm just going to pause.

Interviewer: Okay, we are just resuming interview ID 0014. And just to ask, I don't think I got the answer. Do you believe you were in an emergency department during COVID-19?

Participant 14: No.

Interviewer: No? Have you interacted with the overdose outreach team or another outreach team during COVID?

Participant 14: I interact with them so often that it's a, yeah.

Interviewer: You have? Did you experience any change in the way you were treated or the services you were able to receive from the outreach team?

Participant 14: Pardon?

Interviewer: So you said you have interacted with the outreach team during COVID. Did you notice any change in the way you were treated?

Participant 14: Yeah. An absolute disregard towards my well being and and disrespectfully just dehumanization, disproportionately um you know, I have never been treated like this. So it's an adjustment that I don't think that anybody should have to make, you know what I mean.

Interviewer: Okay, and I just want to confirm, are you talking about the emergency department? Are you talking about outreach? Are you talking about other people here?

Participant 14: I'm talking about everybody.

Interviewer: Okay, just everybody. Okay. So the question is just specifically about the outreach team did you experience any change in the way they treated you? compared to before COVID?

Participant 14: I honestly I'm really grateful for everything that is come of the [inaudible] from the misfortunate last few years and I'm very, very fortunate to have survived all of the attacks and the constant, just all of the accidents I experienced. Not many people would have made it through. But fortunately I have people in my life that work really hard to protect me and therefore I must say that the outreach team was, I think that we need to work on having advocacy, better advocates.

Interviewer: For the outreach? Or for yourself?

Participant 14: Well for anybody who requires an advocate.

Interviewer: Okay. And you mentioned, so you had contact with the outreach team. Did you notice any change in the services you were able to receive from them?

Participant 14: Pardon?

Interviewer: So you mentioned that during COVID, you have had contact from the outreach team. So compared to before COVID was there any difference in the services you were able to receive?

Participant 14: Um, sorry. I'm not sure.

Interviewer: Okay, is there anything you think they can do better?

Participant 14: Sorry

Secondary interviewer (JH): That's okay. Can you just repeat the question, please? Yeah.

Interviewer: Yeah. Is there anything you think the outreach team could do better?

Participant 14: Um, that's that's not my, that's not my place to say.

Interviewer: Okay. Did anyone reach out to you during COVID about getting prescribed a supply of safe opioids?

Participant 14: Yup

Interviewer: Okay, do you remember who reached out to you?

Participant 14: It would of been me reaching out to you guys.

Interviewer: It was the outreach team?

Participant 14: No, it was me, reaching out to you guys.

Secondary interviewer (JH): One more time? Sorry.

Interviewer: Can you-, do you remember who reached out to you about getting prescribed a supply of safe opioids?

Participant 14: Nope.

Interviewer: Did anyone reach out to you?

Participant 14: No.

***Participant 15***

Interviewer: So to start with what is your understanding of COVID-19?

Participant 15: It's from people eating bats. To the best of my knowledge it's a virus that is like affects animals and then mutates into something that humans can contract. So there's a link to swine flu and other shit like that.

Interviewer: What have you learned about how to stay safe during the outbreak?

Participant 15: I’ve honestly given a lot less of a care than I think anybody like comes to it. I don't really do anything.

Interviewer: Okay, how did you learn about COVID-19?

Participant 15: Just I guess paying attention to Donald Trump's poll ratings. I heard about the kung flu, I started listening to it a little bit.

Interviewer: Okay, so through media or through friends?

Participant 15: Yeah, through the news. I suppose.

Interviewer: And can you tell us a bit about what it's been like for you during COVID?

Participant 15: It's been good. I don't know. It's kind of it's kind of nerve wracking that the government gave out money the way that they did. It makes you think that something serious is going on or what not. Other than that, like, woohoo but.

Interviewer: Okay, did you get sick?

Participant 15: Of course not, I never get sick.

Interviewer: Did you get tested?

Participant 15: No.

Interviewer: And did you do anything to keep yourself from getting sick?

Participant 15: No.

Interviewer: Did you notice a change in your community and the way people interacted with each other?

Participant 15: Uh in my community not really no.

Interviewer: Do you think people were scared to help someone having an overdose?

Participant 15: Not sure don't really witness to many of those.

Interviewer: Do you think people were scared to help someone in other ways?

Participant 15: Uh, I've witnessed it, not many but yeah.

Interviewer: Yeah? You've seen that people were scared to help people?

Participant 15: I said not in my community but yeah.

Interviewer: Okay, you've seen it, but it wasn't in your community?

Participant 15: Yeah. Yeah, like people are generally like, excuse my language like scared to shit of one another.

Interviewer: Did COVID-19 affect your ability to get medical care if you needed it?

Participant 15: No, I remember I was by a hospital one time and there was nobody there, it seemed like so, no, I don't think. I don't think it would at all. And it hasn't for me cause I haven't been so.

Interviewer: Okay, were you still able to go to your regular clinic?

Participant 15: Yep.

Interviewer: Did COVID-19 make it more or less likely that you would go to your regular clinic?

Participant 15: Wouldn't affect.

Interviewer: What about, would it make you more or less likely to go to emergency?

Participant 15: Um, wouldn't affect.

Interviewer: And more or less likely to call 911?

Participant 15: Wouldn't affect. Like this COVID wouldn't.

Interviewer: It sounds like it wouldn't affect your decisions about your health care at all?

Participant 15: No, no.

Interviewer: Did it affect your ability to get social support services you needed?

Participant 15: No. It's been really good that way.

Interviewer: Did you need a shelter during COVID-19?

Participant 15: No.

Interviewer: Did COVID-19 affect your use of drugs?

Participant 15: Yes, it increased it greatly.

Interviewer: You found you were using more?

Participant 15: Yes.

Interviewer: Did you use different kinds?

Participant 15: Nope.

Interviewer: Did you find you are buying more or less drugs at a time?

Participant 15: Of course, I was, I've got thousands of dollars for free

Interviewer: You're buying more?

Participant 15: Yup.

Interviewer: Did it change your ability to use drugs as safely as possible?

Participant 15: Um, protocol has been the same. So.

Interviewer: Okay haven't had-

Participant 15: No safer. no less safe. Yeah.

Interviewer: Okay. Did the social or physical distancing recommendations make you use alone or take other risks?

Participant 15: No. I mean, I don't know, I don't really pay attention to that, unless, unless I can notice I'm like off, being off putting to somebody else by being too close or whatnot. I generally don't care about that.

Interviewer: Have you been to an emergency department during COVID-19?

Participant 15: I did to fetch something. I think it was the Narcan kit or whatnot. That's when I noticed It was like empty in there.

Interviewer: Okay. Did you experience any change in the way you were treated? Or the services you're able to receive?

Participant 15: Just people recoiling when you walk up to them and offering you like hand sanitizer, yeah, pretty much same deal as always.

Interviewer: Have you interacted with the overdose outreach team or other outreach teams during COVID?

Participant 15: No. Yeah, once today and then once like a week ago or whatever to do a different type of questionnaire type of dealio.

Interviewer: Okay, did you experience any changes in the way you were treated or services you were able to receive from the outreach team?

Participant 15: Just like the restriction of personal contact kind of thing like stay here, whatever.

Interviewer: Right. Were you still able to get any services or connections that you wanted?

Participant 15: Yeah.

Interviewer: And did anyone reach out to you during COVID about getting prescribed a supply of safe opioids?

Participant 15: No.

***Participant 16***

Interviewer: So to start with, what's your understanding of COVID-19?

Participant 16: My understanding of it? I don't even like I know it sounds ridiculous, I don't know how to answer that.

Interviewer: Okay, so, do you know, like maybe some of the symptoms -

Participant 16: Like I guess I know about it and educated about it and know the precaution, precautions and like, steps to take to respect others people's space and to like, wear a mask even if you're not sick, but to respect people who maybe have a lower immune system. It's been so stressful like I can't even, I don't even know it's just it's been stressful.

Interviewer: Do you remember how you first learned about COVID?

Participant 16: Yes, I got kicked out of where I was staying. Which was somewhere safe to being on the streets which took a toll on my mental health cause my mental health and like, my anxiety and it triggered a lot of things.

Interviewer: So did you just hear about it like word on the street when you were kicked out?

Participant 16: I, well, I guess it was through [Vancouver housing centre].

Interviewer: That told you about, you know, COVID and what was happening at that time?

Participant 16: Yeah.

Interviewer: And so can you tell us what it's been like for you during COVID? You said it's been pretty stressful.

Participant 16: It's been really stressful. I've been trying to find housing the whole time, and also by going out and going to house viewings and going around to the different shelters and I've been doing that this whole time and still there's like no housing.

Interviewer: Did you get sick during COVID?

Participant 16: I did get sick. Yes, it was before it got really intense, but it was right after being kicked out.

Interviewer: And do you think that it was COVID? Or do you think it was something else?

Participant 16: Uh.

Interviewer: Or you don't know?

Participant 16: Yeah

Interviewer: Yeah, so it's, you know, some of the symptoms are pretty vague. So, did you get tested?

Participant 16: Yeah.

Interviewer: And did you get tested, it sounds like you didn't get tested when you were sick. Maybe afterwards?

Participant 16: Well I had pneumonia. Yeah, I got pneumonia. But I, my immune system cleared all that up pretty good. I eat a lot of yogurt, lots of probiotics.

Interviewer: Okay, and when you got sick you said at the very beginning where you're kind of unsure what it was. What did you do when, when you were sick?

Participant 16: I guess I just tried to stay positive through it all and it was a lot.

Interviewer: What did you do to keep yourself from getting infected?

Participant 16: Frequent handwashing and just kind of distancing but not really. Like me and my partner we would, we would obviously be cautious but we thought the stresses from COVID were gonna be more stressful.

Interviewer: Did you notice a change in your community in the way people interacted with one another?

Participant 16: Absolutely. It seemed like, I don't know. Yeah, everyone, I don't know it just, fear. Lots of stress, lots of mental health and stuff.

Interviewer: Do you think people were scared to help someone having an overdose?

Participant 16: I'm sure people were. Yeah.

Interviewer: Do you think people were scared to help people in other ways too because of COVID?

Participant 16: Sorry, say that-

Interviewer: Do you think because of COVID people were scared to help other people, in other ways not necessarily an overdose but other-

Participant 16: Oh, probably, probably. Yeah.

Interviewer: And is that something you've saw or heard about? Or maybe felt yourself that makes you feel that way?

Participant 16: Yeah, I definitely saw it in the way like people interact and whatnot.

Interviewer: Did COVID affect your ability to get medical care if you needed it?

Participant 16: It did. Yeah.

Interviewer: In what way?

Participant 16: Everything was just a headache. Uh, my mind is overloaded with question questions. Sorry, I don't have.

Interviewer: No worries. We are, we are almost done too, we are on the last page. And a lot of these last questions are just ‘Yes, no’. So they are very easy.

Participant 16: Okay.

Interviewer: Were you still able to go to your regular doctor or clinic if you needed through COVID?

Participant 16: Yeah.

Interviewer: Did COVID make it more or less likely you would go to the doctor?

Participant 16: Yes.

Interviewer: More or less?

Participant 16: Less.

Interviewer: Did COVID make you more or less likely to go to the emergency?

Participant 16: Yes.

Interviewer: And more, or less again?

Participant 16: Less.

Interviewer: And did it make you more or less likely to call 911?

Participant 16: Yes.

Interviewer: Less again?

Participant 16: Yeah.

Interviewer: And did it, how did it affect your decisions about when and where you needed health care?

Participant 16: Sorry, say that again.

Interviewer: How did COVID-19 affect your decisions about when and where you went and got health care? Or it sounds like you just got, tried to get less health care?

Participant 16: Like, I guess like I just started methadone yesterday. I've been trying to get clean through all of it, but the stress like the anxiety and like I spent, I've spent $2,000 on like a deposit which ended up being a scam. I even went and viewed the house and all of that, which was stressful, because something happened with my bank. And so, money that I was saving up to kind of help, like be able to go towards housing and emergency funds and like food I didn't have access to so it kind of ended up being um.

Interviewer: Just kind of slowed down everything?

Participant 16: Yeah.

Interviewer: Did COVID-19 affect your ability to get social support services you needed? You mentioned it's been pretty hard for you to get a shelter. Is there any other support services that it's affected?

Participant 16: No, I think like, just that yeah.

Interviewer: So this question is, did you need a shelter during COVID-19, I get from you the answer is yes?

Participant 16: Yes.

Interviewer: And were you able to get a shelter at all?

Participant 16: I was yes.

Interviewer: And how did that go? Was it easier to get it or did it take much longer than usual?

Participant 16: No, it the, it wasn't, it wasn't too bad. Except I, I wouldn't, like I didn't get much sleep because of the high stress and like, having really bad anxiety, and all the emotions and other people's stresses.

Interviewer: So you were able to get the shelter okay, but it didn't alleviate all the problems, obviously?

Participant 16: Yeah.

Interviewer: Did COVID-19 affect your use of drugs?

Participant 16: Uh I've cut down a lot.

Interviewer: Okay, so did you find you were using less because of COVID?

Participant 16: Yeah

Interviewer: Did you find you were using different kinds?

Participant 16: Yes.

Interviewer: And did you find you were buying more or less drugs at a time? Or no change?

Participant 16: I guess. More but less, if that makes sense. I was using less but like had a higher tolerance I guess.

Interviewer: Did it change your ability to use drugs as safely as possible?

Participant 16: Yes.

Interviewer: And in what way?

Participant 16: Being kicked out of a safe place to be on the streets by myself at 25. It was definitely, it's not easy. Like it's not easy.

Interviewer: Did you find any difficulty getting into an overdose prevention site or getting safe supplies if you needed them?

Participant 16: What's that?

Interviewer: Because of COVID, did you find it difficult getting any safe supplies you may have needed?

Participant 16: Yeah. With everything being slower.

Interviewer: And did the social or physical distancing recommendations make you use alone? Or take other risks?

Participant 16: Ah, yeah.

Interviewer: You find you were using alone more?

Participant 16: Yeah.

Interviewer: Have you been to an emergency department during COVID-19?

Participant 16: Yes.

Interviewer: Did you notice any changes?

Participant 16: No.

Interviewer: Were you still able to get all the services that you wanted to receive?

Participant 16: Yeah, I guess yeah.

Interviewer: And was there any change in the way you were treated?

Participant 16: I just found people not like taking me seriously, like taking me serious. But like understandable though with all this stress, and like so much respect to the people who, you know, got up and went to work and um.

Interviewer: Have you interacted with the overdose outreach team or other outreach teams during COVID?

Participant 16: No. I haven't.

Interviewer: And did anyone reach out to you during COVID about getting prescribed a supply of safe opioids?

Participant 16: Uh no.

***Participant 17***

Interviewer: So to start with what is your understanding of COVID-19?

Participant 17: It's not very good. Um. I don't know where it came from [inaudible]

Interviewer: What have you learned about how to stay safe during the outbreak?

Participant 17: I fell self taught by myself. They say to wash your hands a lot. There's a lot, there is different methods of keeping clean now I seen on tv, I don't watch very much. I don't know.

Interviewer: And how did you first learn about COVID-19?

Participant 17: Um, I actually [inaudible]

Interviewer: Sorry, how-

Participant 17: Um, he was just uh.

Interviewer: How did you first hear about COVID? Was it the news or was it in doctors, or word of mouth?

Participant 17: News, uh word of mouth. It was by someone. It wasn't the news. It was by someone. Probably by mom, yeah, my mom always updates me on everything.

Interviewer: So can you tell us what it's been like for you during COVID?

Participant 17: Yeah, I think most people answer this when they feel, I feel centered out more, cause it's not just two and a half things I have going on there's three and a half things now. I want to stop adding these things that are making me feel- [inaudible]. Um.

Interviewer: Has there been any impact on your life? Because of COVID?

Participant 17: No.

Interviewer: Did you get sick?

Participant 17: No

Interviewer: Did you get tested?

Participant 17: No.

Interviewer: And what did you do to keep yourself from getting sick?

Participant 17: I drank water.

Interviewer: You drank water?

Participant 17: Yeah lots of water.

Interviewer: Okay, did you do anything else to keep yourself from getting sick?

Participant 17: Um, I take my shoes off, and blankets off. The simple stuff.

Interviewer: Did you notice a change in your community in the way people interacted with each other?

Participant 17: Here? Yeah. Oh, definitely. We all, we all got offended at some point. It's understandable I guess, when the staff members started to wear masks and stuff. They're just doing what they're mandatory told to do by their boss. Right.

Interviewer: Okay, that created a little bit of tension?

Participant 17: Oh sure it did. Oh yeah, totally.

Interviewer: Do you think people were scared to help someone having an overdose because of COVID?

Participant 17: Yeah.

Interviewer: Do you think people were scared to help someone in other ways too, because of COVID. Not necessarily an overdose, but if someone needed help?

Participant 17: Yup.

Interviewer: And is this something you saw or heard about that you think people were scared?

Participant 17: Yep.

Interviewer: Was that you saw?

Participant 17: I saw, yeah.

Interviewer: And what did you see?

Participant 17: I saw, uh. I saw, just people, sorry, can you repeat-

Interviewer: What have you seen that make makes you think that people were scared to help someone?

Participant 17: Oh, oh yeah, no just they see, sorry I do have an answer for that. They see the sores on their face, if they're flailing around, you know the flail. That's scary, it makes me angry when I guys [inaudible] as big of shot as I did and it did that to you? You shouldn't even be doing this man. But it's the addiction. Yeah, I could be that too, um, just look I guess. Yeah, they see them flailing. They usually carry, fortunately, some some actually make the other ones look so much worse. That's unfortunate too.

Interviewer: Did COVID-19 affect your ability to get medical care if you needed it?

Participant 17: Yeah, yeah

Interviewer: Yeah, And in what way?

Participant 17: Well, it uh, [inaudible] take a long to go away, got sores on your body and it never properly heals.

Interviewer: So were you still able to go to a doctor or clinic if you need it?

Participant 17: Oh yeah.

Interviewer: Did COVID make it more or less- sorry?

Participant 17: at the modular [pen?]

Interviewer: And so you said that COVID did affect your medical care or your ability to get medical care. So in what way did it affect your ability to get medical care?

Participant 17: It was so difficult, because the person I go to with a lighter. This is like 100% my fault too, I see my part in it.

Interviewer: Sorry the place you go to-

Participant 17: Well I chose a place that was um I guess it was on [Vancouver street] or something, it was day time, and uh I wanted to just get, I wanted to get some beer halfway home, I wanted to get some beers to drink right then too. I started drinking right away.

Interviewer: Sorry, could I, I just want to make sure the audio is good. Um, so you said that COVID affected your ability to get medical care. And so in what way did it affect that?

Participant 17: Well, the emergency would be so busy. They can't attend to people as much as it, because of all the cleaning procedures and behind glass and stuff and any addition, any additional thing the hospital would like to do other than search for victims of it is, um they've done so much work on the hospitals and stuff. And transport and stuff like that. I’m thinking, ‘Where's the money come from?’

Interviewer: Did COVID make it more or less likely that you would go to the doctor? or no change?

Participant 17: No change.

Interviewer: Did it make it more or less likely that you would go to the emergency?

Participant 17: More or less likely that I'd go to the emergency?

Interviewer: Yeah, because of COVID?

Participant 17: Yeah, possibly.

Interviewer: More or less?

Participant 17: More

Interviewer: Did COVID make it more or less likely that you would call in 911?

Participant 17: If someone was sick?

Interviewer: Yeah.

Participant 17: Oh, for sure. Yeah, even nipping it in the butt a little earlier just in case right?

Interviewer: Did it make you more or less likely would call 911?

Participant 17: It made me more likely I would [inaudible].

Interviewer: And how did COVID affect your decisions about when and where you needed health care?

Participant 17: Um, sorry, one more time?

Interviewer: How did COVID-19 affect your decisions about when and where you needed health care?

Participant 17: Um.

Interviewer: Did it affect if you needed, if you needed any medical care, did it change where you went or when you went?

Participant 17: I guess it could, I never really focused on that, no.

Interviewer: Did COVID affect your ability to get social support services you needed?

Participant 17: I think so.

Interviewer: And in what way?

Participant 17: [inaudible] the right person

Interviewer: Sorry what was that?

Participant 17: I couldn’t get the right question out there, what I was looking for.

Interviewer: Okay, you were seeking some-

Participant 17: Yeah, I couldn't get the message across the person.

Interviewer: Okay, and what were you, what were you trying to get and you weren't able to receive?

Participant 17: I can't remember exactly.

Interviewer: Okay. Did you need a shelter during COVID?

Participant 17: Yeah, I did.

Interviewer: You did? And how did that go? Did you get a shelter?

Participant 17: Yeah. I stayed at the, what’s that called down there, up [Vancouver street].

Interviewer: Was it, was it easy to get a shelter?

Participant 17: Um, it's easy as usually [inaudible]

Interviewer: Did COVID-19 effect you use of drugs?

Participant 17: Yeah it did.

Interviewer: Did you find you were using more or less?

Participant 17: I tried to use more right away [inaudible] to right away. Cause the flap was actually smaller than I thought.

Interviewer: Sorry, that was you, you were using more?

Participant 17: Oh yeah, I guess more.

Interviewer: Do you find that you're using different kinds because of COVID?

Participant 17: Uh not because of COVID just because there's different kind available right now.

Interviewer: Okay. Did you find you were buying more or less drugs at a time because of COVID?

Participant 17: No I was buying um.

Interviewer: No change?

Participant 17: No change.

Interviewer: Did COVID change your ability to use drugs as safely as possible?

Participant 17: Yeah. It taught me to be cleaner about it and consider other people more, what they might see when they walk by or just the fact [inaudible]

Interviewer: Sorry can you just repeat the answer, did COVID change your ability to use drugs as safely as possible?

Participant 17: Yeah, it made it better, more awareness to be a safer drug user is what I am saying.

Interviewer: Oh, it made it more-

Participant 17: I wanted to clean up because I have a daughter you know, very young daughter and I picture if I throw a rig down the ground that she can come along play and pick it up. So that come plays in my head every time I see a rig, or I'm about to throw it in the garbage or something, ‘no, find a box, put it properly’. And then taking care of myself like the germs and stuff, I use alcohol swipes, all my alcohol swabs, I use a lot of them.

Interviewer: Okay, so COVID and all of the cleaning recommendations made you actually be more safe?

Participant 17: That's right, more safe. Because of the possible uh because germs can be contracted easier with the COVID.

Interviewer: That is a very interesting point. Did you have any difficulty getting safe supplies or getting into an overdose prevention site?

Participant 17: Um, no.

Interviewer: And did the social physical distancing recommendations make you use alone or take other risks you might not usually have taken?

Participant 17: um, [inaudible]

Interviewer: Did you find you were using more um sorry, did you find because of COVID you were using alone more?

Participant 17: Um

Interviewer: Or was there no change?

Participant 17: No, no I use more alone. And I hate being alone.

Interviewer: Okay, but you find that the social distancing recommendations have made you use alone?

Participant 17: No, no, no that didn't have to do with why I was using alone. It's just that it's illegal. To have your full arm span right.

Interviewer: Have you been to the emergency department during the COVID-19?

Participant 17: Yep.

Interviewer: And did you notice any changes in the emergency department?

Participant 17: Yeah, there's a lot of curtains and stuff. Different routines, different ways to get rooms now, you know, the safest ways right. [inaudible] for the hospital and they put a plan together and it seems to be working.

Interviewer: Okay. You say it seems to be working. Did you experience any change in the way you were treated? Or the services you were able to receive?

Participant 17: No, I find there's more help at the counter, when they, I was called up and I was called up pretty quickly.

Interviewer: Have you interacted with the overdose outreach team or other outreach teams during COVID?

Participant 17: Yeah.

Interviewer: And did you experience any change in the way you were treated? Or the services you were able to receive by the outreach team?

Participant 17: Yeah, I would say so.

Interviewer: And in what way?

Participant 17: Um, [inaudible] another garbage bag, I got to take, haul some garbage out with. Sorry.

Interviewer: So you said that you had interacted with the outreach team during COVID-19?

Participant 17: Yes.

Interviewer: Did you experience any change in the way you're treated, because of COVID?

Participant 17: I don't know. Because, honestly, they do, half the time when I do notice a change or something or like that, COVID doesn't come to my mind. It doesn't come to my mind with a lot of a lot of what these questions are asking me or being asked is and we're talking about my addiction, right. And they don't put the two together very much. It's there, it is raging around you, everyone's wearing masks and everything. I just feel that there's not much more to be said about the COVID once everyone's taking the precautions that they're supposed to take.

Interviewer: Okay, so when you were with the outreach team, COVID didn't change the way you were treated or any services you're able to receive?

Participant 17: No. Not that I know of.

Interviewer: And did anyone-

Participant 17: But now I’m starting to wonder, when you ask me these, but are they though? Are they being affected? I wonder if they are, I don't know.

Interviewer: If you're not immediately thinking of any changes, maybe not? But if you notice anything in the future, you're welcome to contact [outreach team worker] and let, let him know. Did you, did anyone reach out to you during COVID about getting prescribed a supply of safe opioids?

Participant 17: Not really. No.

***Participant 18***

Interviewer: So, to start with what is your understanding of COVID-19?

Participant 18: It sucks.

Interviewer: It sucks. What, do you know a lot about what it is and the symptoms?

Participant 18: It's a ruse probably started by Trump and some Republicans to cover up the total dissolvement of their relationship with China so that they can come in later and bomb them and rape them for all their shit. And they're calling it the Wuhan virus. They can be like oh [inaudible] holy shit worldwide and take them out. That's why, that's why I think from the beginning I was like wow, this is the reason for them to like cut China out of everything and like be like [inaudible] china to the world guys, huh, c'mon, fuck them. So yeah, I think its bullshit and I think it's just like I said a ruse. I think it's just a ruse like most major problems are that are global or international or whatever you know that gets headlines. I think a lot of them are, well if not doctored, doctored by specifically the United States.

Interviewer: What have you learned how to stay safe during the outbreak?

Participant 18: Safe from what?

Interviewer: Have you done anything to, anything differently in your life to stay safe?

Participant 18: Safe from what?

Interviewer: Like COVID-19, and to be infected?

Participant 18: I wear a mask

Interviewer: Anything else?

Participant 18: I don't hang around people, but I don't do that anyway. So it's like, not really not a lot of change for me.

Interviewer: How did you, how did you first learn about COVID-19?

Participant 18: Someone told me about it.

Interviewer: And so can you tell us a bit what it's been like for you during COVID?

Participant 18: As far as the COVID itself?

Interviewer: Just any impact on your life?

Participant 18: From COVID?

Interviewer: Yeah.

Participant 18: Well, it definitely made me a lot harder to take pictures and identify all the gang stalkers that are following me around. That's for sure. And the one dude that's supposed to be this serial killer that everyone's been taking pictures of me for. He's pretty hard to keep track of now too cause you got these other brown dudes with masks that look like him walk around, this I don't know other, this other dude, he's got a twin or something like that and he's really creepy and keeps, it's making it hard for me to find these people that are trying to hurt me, that's for sure. Because they can all wear masks now. So it makes it easy for them to just like other faces when I try and take pictures.

Interviewer: Okay, did you get sick?

Participant 18: Of what, the gang stalkers?

Interviewer: With COVID?

Participant 18: Sick of it?

Interviewer: No. Like, did you get infected? Did you get-

Participant 18: Oh no.

Interviewer: Did you get tested for COVID? Have you gone like, you know, the swab test up the nose?

Participant 18: No, because I had no reason to think that I was sick.

Interviewer: Okay, did you notice a change in your community in the way people interacted with each other?

Participant 18: Oh, for sure.

Interviewer: And in what ways?

Participant 18: Oh, it finally gave all that and, you know, entitled people in this city reason to feel more entitled. Like ‘See, I told you. I told you to stay the fuck over there'. Just makes the dicks more, more overheaded with their ignorance, I guess.

Interviewer: Do you think people were scared to help someone having an overdose?

Participant 18: Oh, definitely. People are scared to do that, no matter what. Not even scared, they just don't care literally.

Interviewer: Do you think COVID made people more scared?

Participant 18: Yes. For sure people are more scared to do anything. Just like I said COVID like instilled fear in everyone, that's not even like as big as it should be I think.

Interviewer: Do you think people where scared to help others in other ways, too, not even just an overdose?

Participant 18: Oh, I know they are. I touched the glass in the store. and people were like [raised voice] ‘Excuse me, sir’. I get yelled at like, jesus. Sorry.

Interviewer: Did COVID-19 affect your ability to get medical care if you needed it?

Participant 18: I don’t, actually yes, yes, it did. But in my opinion, for myself at least, I kind of wish that it was still going the way it was because I would really like to not have to go to the doctor when I need stuff. Just going over the phone, that was pretty rad.

Interviewer: Okay, so it affected it in the sense you had to do it over the phone, but you were still able to get all the services?

Participant 18: Oh yeah, I got everything I needed, but I didn't even have to leave my house which is really nice.

Interviewer: And this one was, so were you still able to go to your regular doctor or clinic? You said yes it just changed the method. Um did COVID-19 make it more or less likely that you would go to the doctor?

Participant 18: Less.

Interviewer: Did it make it more or less likely go to the emergency?

Participant 18: Less. I don't know, it didn't do any, it didn't change my, I don't, no more.

Interviewer: No change is an option.

Participant 18: Yeah, no change, yeah.

Interviewer: And did it make it more or less or no change that you would call 911?

Participant 18: No change

Interviewer: And how did it affect your decisions about when and where you needed health care?

Participant 18: Uh.

Interviewer: If you need any health care, did it change where and when you went?

Participant 18: Well it just changed like I said where because I did it over the phone, right so, and when I guess because I could do it in the morning right away or I'd have to do on specific days my doctors in so that's another thing.

Interviewer: Did COVID-19 affect your ability to get social support services you needed?

Participant 18: Yes.

Interviewer: And in what way?

Participant 18: A lot of places were shut down and some still are shut down, and a lot of them actually shut down for good.

Interviewer: And did you need a shelter during COVID-19?

Participant 18: I would like one that's not mine, but I guess technically no.

Interviewer: Did COVID-19 affect your use of drugs?

Participant 18: Yes.

Interviewer: Did you find you were using more or less?

Participant 18: More.

Interviewer: Did you find you were using different kinds because of COVID?

Participant 18: I don't, I guess maybe because of just the market influx of, and you know, prices and everything and totally just yeah definitely got me using more drugs, other drugs.

Interviewer: Did you find you are buying more or less drugs at a time?

Participant 18: Yes.

Interviewer: More or less?

Participant 18: More.

Interviewer: And what was the reason? Was there any specific reason?

Participant 18: Well there was a lull in one drug and just I don't know, I guess, relationships I built recently. There's more so of just peer pressure influence by my peers more so than just my decision, I guess.

Interviewer: And did COVID-19 change your ability to use drugs as safely as possible?

Participant 18: Well I think that's obvious, yes. Because now you got this pandemic going around. So I think it changes every body's ability to do anything as safely as possible when there’s a pandemic.

Interviewer: Did you have difficulty getting safe supplies, maybe?

Participant 18: Yep.

Interviewer: Um, did you have difficulty getting into an overdose prevention site?

Participant 18: I don't know.

Interviewer: Okay. And did the social or physical distancing recommendations make you use alone? Or take other risks you may not usually have taken?

Participant 18: Uh.

Interviewer: Did you find you're using alone more because of COVID?

Participant 18: I guess yeah, I guess. Yeah, that's true.

Interviewer: Have you been to an emergency department during COVID-19?

Participant 18: Yes.

Interviewer: And did you notice any changes?

Participant 18: Yeah there was no one there. It was awesome.

Interviewer: Okay, did you experience any change in the way you were treated or services you were able to receive?

Participant 18: Yeah, I was actually treated really well, and it went super quick and super smooth and so I was pretty stoked on that cause there was literally no one else there. I went twice, actually. And one time there was three other people, one left, and the other time, oh I went three times sorry. Twice there was no one else there. And one time there was three other people.

Interviewer: Okay, and you were able to get everything you wanted and quicker, you said?

Participant 18: Yeah, it was awesome. And with better care, like, you know, nicer, nurses and, and whatnot because they weren't, you know, swamped with a bunch of people demanding things. And so I think they were just a lot more relaxed and you know, easygoing with it.

Interviewer: Awesome that that's really good feedback there. Have you interacted with the overdose outreach team or other outreach teams during COVID-19?

Participant 18: No, I haven't. No.

Interviewer: And did anyone reach out to you during COVID about getting prescribed a supply of safe opioids.

Participant 18: Yeah they did

Interviewer: And so who reached out to you?

Participant 18: My doctor,

Interviewer: And were able to get a supply?

Participant 18: Somewhat.

Interviewer: Somewhat? Was it helpful?

Participant 18: For a bit, kind of. Actually, I don't know, I think, no, it was actually, it actually in the end it made it worse because the clinic I go to the doctors rotate and also this whole other thing with my doctor. Anyway, what happened was basically my doctor was giving them to me at a very high amount and then they just got cut off like BAM and I wasn't wanting to go off on anything either. And I literally I got cut off on my whole agonist and everything at the same time so I was like horribly, horribly ill. And so the next thing you know, I'm just like back to using a whole bunch again, the one way I could cope with it. It was just really messed up, it sucked.

Interviewer: Okay, so it's helpful at the time, but then ended up stopping and then it was almost worse?

Participant 18: And then yeah, no it made it way worse because I've had to, just that kind of like, was a catalyst of, you know, a whole bunch of other events happening in my life that led to me losing my methadol prescription, which I think is the stupidest thing that you guys do that here. And so that, you know, from there, I went back to using and I'm not anything now.

***Participant 19***

Interviewer: So to start with what is your understanding of COVID-19?

Participant 19: I don't know.

Interviewer: Do you know what it is and what the symptoms are?

Participant 19: No.

Interviewer: No? Have you learned anything about how to stay safe during COVID?

Participant 19: No.

Interviewer: And you remember when you first heard about COVID, maybe not about what it is, but just hearing you know the word COVID and that there's this pandemic happening, do you remember how you first heard about it?

Participant 19: I don't remember, I can't remember.

Interviewer: Was it, do you think maybe a doctor or friends, word on the street, media?

Participant 19: Yeah I think it was, yeah the word on the street, yeah.

Interviewer: Can you tell us what it's been like for you during COVID?

Participant 19: Everything just changed.

Interviewer: What kind of changes happened?

Participant 19: You had to wear masks, the two meters, you know.

Interviewer: Has there been any impact on your life because of COVID?

Participant 19: No,

Interviewer: Did you get sick?

Participant 19: No.

Interviewer: Did you get tested?

Participant 19: No.

Interviewer: And what have you done to keep yourself from getting infected?

Participant 19: I don't know.

Interviewer: Have you done anything to keep yourself from getting sick?

Participant 19: Nope.

Interviewer: No?

Participant 19: No.

Interviewer: Did you notice a change in your community in the way people interacted with each other?

Participant 19: Yeah kinda.

Interviewer: And what kind of changes did you see?

Participant 19: Where we have to wear masks and stuff.

Interviewer: Okay. Do you think people were scared to help someone having an overdose, because of COVID?

Participant 19: Yeah.

Interviewer: Do you think people were scared to help people in other ways because of COVID?

Participant 19: Yeah.

Interviewer: Yeah?

Participant 19: Yep.

Interviewer: And is that something you saw or heard about that makes you think that?

Participant 19: I don't know.

Interviewer: What makes you say that people were scared to help other people because of COVID?

Participant 19: I don't know.

Interviewer: Okay, did COVID affect your ability to get medical care if you needed it?

Participant 19: Um kinda yeah.

Interviewer: And in what way?

Participant 19: I don't know.

Interviewer: Were you still able to go to your regular doctor as frequently as you wanted?

Participant 19: Yep

Interviewer: Did COVID-19 make it more or less likely you would go to the doctor? or no change?

Participant 19: No change, yeah.

Interviewer: Did it make it more or less likely you would go to the emergency?

Participant 19: Pardon?

Interviewer: Did COVID-19 make it more or less likely that you would go to the emergency?

Participant 19: Uh less.

Interviewer: Less? And did it make it more or less likely you would call 911?

Participant 19: Uh no.

Interviewer: And how did it affect your decisions about when and where you needed health care?

Participant 19: I don't, I'm not too sure.

Interviewer: Did it change at all about when you went and sought health care or where you went to get health care?

Participant 19: Pardon?

Interviewer: Did COVID change when you went out to get health care or where you would go to get health care? Or no change?

Participant 19: Changed a little

Interviewer: Changed a little bit? And in what way? How did it change it?

Participant 19: The way, the way how we're sitting down right now. We have to be like two meters away and stuff like that, wearing masks during meetings. Cause sometimes it's hard to breathe under, under these masks, hey.

Interviewer: Did COVID affect your ability to get social support services you may have needed?

Participant 19: I don't, no, I don't know.

Interviewer: Okay, did you need a shelter during COVID-19?

Participant 19: Yeah.

Interviewer: And were you able to get one?

Participant 19: No, but I'm in one now.

Interviewer: Okay, so you eventually were able to get one?

Participant 19: Yeah.

Interviewer: And did you find that the process was easier or harder because of COVID? Did you find it was easy to get a shelter?

Participant 19: Yeah.

Interviewer: Just as easy as before COVID?

Participant 19: Yeah.

Interviewer: Or a bit harder? Just as easy?

Participant 19: Just, just as easy.

Interviewer: Did COVID-19 affect your use of drugs?

Participant 19: No.

Interviewer: Did you find you are using more or less? Just going to make sure this is recording.

Participant 19: Hey can you say that again?

Interviewer: Yeah. Did you find you're using more or less drugs because of COVID or during COVID?

Participant 19: A little bit of both.

Interviewer: Okay, so depending on the kind?

Participant 19: Yeah.

Interviewer: Okay. Did you find you're using different kinds during COVID?

Participant 19: Yeah.

Interviewer: Did you find you're buying more or less drugs at a time?

Participant 19: Um, more.

Interviewer: Okay, was there any reason for buying more?

Participant 19: Just so I don't have to keep on running back.

Interviewer: So don't have to keep going back?

Participant 19: Yeah.

Interviewer: Okay. Did COVID change your ability to use drugs as safely as possible?

Participant 19: Nope.

Interviewer: Did you, you have any difficulty getting safe supplies?

Participant 19: No.

Interviewer: Did you have any difficulty getting into overdose prevention sites?

Participant 19: No.

Interviewer: Did this social or physical distancing recommendations, make you use alone or take other risks you may not have usually taken?

Participant 19: Pardon?

Interviewer: Did the social or physical distancing recommendations, so the recommendation, you know, to stay two meters apart, did that make you use alone more often? Or take other risks?

Participant 19: I don't know.

Interviewer: You don't know? You didn't notice any change?

Participant 19: No.

Interviewer: Have you been to an emergency department during COVID?

Participant 19: No.

Interviewer: Have you interacted with the overdose outreach team or other outreach teams during COVID?

Participant 19: No.

Interviewer: And did anyone reach out to during COVID about getting prescribed a supply of safe opioids?

Participant 19: Yeah.

Interviewer: Yeah? Do you remember who reached out to you?

Participant 19: Pardon?

Interviewer: Do you remember who reached out to you and told you about getting prescribed supply of safe opioids?

Participant 19: No.

Interviewer: You don't remember?

Participant 19: I can't remember, no.

Interviewer: Okay. But someone did reach out to you?

Participant 19: Yeah.

Interviewer: Okay. Were you able to get a supply?

Participant 19: Yeah. I think so, yeah.

Interviewer: Did you find it was helpful?

Participant 19: Yeah.
